# Analysis of the Genome of a Korean Isolate of the *Pieris rapae* Granulovirus Enabled by Its Separation from Total Host Genomic DNA by Pulse-Field Electrophoresis

**DOI:** 10.1371/journal.pone.0084183

**Published:** 2013-12-31

**Authors:** Yong Hun Jo, Bharat Bhusan Patnaik, Se Won Kang, Sung-Hwa Chae, Seunghan Oh, Dong Hyun Kim, Mi Young Noh, Gi Won Seo, Heon Cheon Jeong, Ju Young Noh, Ji Eun Jeong, Hee Ju Hwang, Kisung Ko, Yeon Soo Han, Yong Seok Lee

**Affiliations:** 1 Department of Life Science and Biotechnology, College of Natural Sciences, Soonchunhyang University, Asan, South Korea; 2 Division of Plant Biotechnology, College of Agriculture and Life Sciences, Chonnam National University, Gwangju, South Korea; 3 Hampyeong County Insect Institute, Hampyeong County Agricultural Technology Center, Hampyeong, South Korea; 4 Department of Medicine, Medical Research Institute, College of Medicine, Chung-Ang University, Seoul, South Korea; 5 GnC Bio Company Limited, Daejeon, South Korea; University of North Texas, United States of America

## Abstract

**Background:**

Most traditional genome sequencing projects involving viruses include the culture and purification of the virus particles. However, purification of virions may yield insufficient material for traditional sequencing. The electrophoretic method described here provides a strategy whereby the genomic DNA of the Korean isolate of *Pieris rapae* granulovirus (PiraGV-K) could be recovered in sufficient amounts for sequencing by purifying it directly from total host DNA by pulse-field gel electrophoresis (PFGE).

**Methodology/Principal Findings:**

The total genomic DNA of infected *P. rapae* was embedded in agarose plugs, treated with restriction nuclease and methylase, and then PFGE was used to separate PiraGV-K DNA from the DNA of *P. rapae*, followed by mapping of fosmid clones of the purified viral DNA. The double-stranded circular genome of PiraGV-K was found to encode 120 open reading frames (ORFs), which covered 92% of the sequence. BLAST and ORF arrangement showed the presence of 78 homologs to other genes in the database. The mean overall amino acid identity of PiraGV-K ORFs was highest with the Chinese isolate of PiraGV (∼99%), followed up with *Choristoneura occidentalis* ORFs at 58%. PiraGV-K ORFs were grouped, according to function, into 10 genes involved in transcription, 11 involved in replication, 25 structural protein genes, and 15 auxiliary genes. Genes for *Chitinase* (ORF 10) and *cathepsin* (ORF 11), involved in the liquefaction of the host, were found in the genome.

**Conclusions/Significance:**

The recovery of PiraGV-K DNA genome by pulse-field electrophoretic separation from host genomic DNA had several advantages, compared with its isolation from particles harvested as virions or inclusions from the *P. rapae* host. We have sequenced and analyzed the 108,658 bp PiraGV-K genome purified by the electrophoretic method. The method appears to be generally applicable to the analysis of genomes of large viruses.

## Introduction

Baculoviruses represent a diverse group of viruses with covalently closed, double-stranded, circular, supercoiled genomes, with sizes varying from 80 to 180 kb, encoding between 90 and 180 genes. The DNA genome is packaged in rod-shaped nucleocapsids that are 230–385 nm in length and 40–60 mm in diameter. The virions occur in two types- occluded virions (ODV) and budded virus particles (BV). Baculoviridae are divided into four genera, *Alphabaculovirus* [lepidopteran-specific nuclear polyhedrosis virus (NPVs)], *Betabaculovirus* [lepidopteran-specific granulosis virus (GVs)], *Gammabaculovirus* (hymenopteran-specific NPVs) and *Deltabaculovirus* (dipteran-specific NPVs) [1], [2]. Viruses belonging to the order Hymenoptera contain the smallest genomes, at >80 kb, which has been explained as a result of their restricted life cycle, confined to replication in insect gut cells [3]. Group I alphabaculoviruses cluster ∼130 kb, whereas Group II shows a high degree of diversity, varying from ∼130 to 170 kb. The larger genomes of the Group II alphabaculoviruses can be attributed to a combination of repeated genes that are not found in the smaller genomes. This is in contrast to the betabaculoviruses genomes, varying from 101 kb in the case of *Plutella xylostella* granulovirus (PlxyGV) [4] to 178 kb in *Xestia c-nigrum* granulovirus (XecnGV) [5]. Despite the large difference in gene content in betabaculovirus genomes, as reflected in this range of sizes, their genomes are surprisingly collinear, compared with alphabaculoviruses, which show a high degree of variation [6], [7]. The first dipteran-specific deltabaculovirus, the *Culex nigripalpus* nucleopolyhedrovirus (CunniNPV), was isolated and sequenced from the mosquito *Culex nigripalpus* [8]. A phylogenetic analysis showed its distinctive form, making it a member of a new genus within the family Baculoviridae [9]. Compared to alphabaculoviruses family members, betabaculoviruses have been investigated to a lesser degree, because of the limitations of permissive cell lines [10]. Currently, 60 complete genomes are known in the Baculoviridae family; 45 genomes from NPV (41 alphabaculoviruses, 3 gammabaculoviruses, and 1 deltabaculovirus), 14 genomes from GV, and 1 unclassified *Hemileuca* sp. NPV (http://www.ncbi.nlm.nih.gov/genomes/GenomesGroup.cgi?taxid=10442).

The small cabbage white butterfly, *Pieris rapae* (*P. rapae)* is a serious pest of cultivated cabbages and other mustard family crops worldwide. A serious infestation can lead to the death of the plant due to reduced photosynthesis. *P. rapae* granulovirus (PiraGV) infects *P. rapae* in nature and functions as an important biological agent in controlling the population of *P. rapae* in the ecosystem. Although PiraGV is now a registered biocontrol agent for the control of *P. rapae*, research on the genetic and molecular information of the virus is still limited, apart from a recent study on occlusion-derived virus (ODV)-associated proteins of the betabaculovirus [11]. Sequencing of the complete genome of the Chinese isolate of *P. rapae* granulovirus (PiraGV-C) showed a size of 108,592 bp and predicted 120 open reading frames (GenBank, GQ884143) [12]. Although sequencing efforts have been significant, more detailed information about a wide range of isolates inhabiting different geographical regions would provide a more comprehensive overview of baculoviruses and further establish their candidature as pest control agents.

This study is unique, as we have taken advantage of the large-sized genome and high titer of infection of *P. rapae* granulovirus (Korean isolate) to purify the viral genome away from host DNA by pulse-field gel electrophoresis. The viral DNA is recovered in amounts sufficient for its classical genome sequencing. The procedure requires less starting material than would be necessary if starting with the purification of virus particles from inclusion bodies. The genome sequence produced in this work was through a subcloning approach, without recourse to the use of automated high-throughput next-generation sequencing (NGS) technology.

## Materials and Methods

### Separation of Nuclei from *P. rapae*

Larvae of *P. rapae* were obtained from a mass rearing facility at Hampyeong Insect Institute (Hampyeong, Korea) and were reared in the laboratory on kale leaf at 25±3^o^C with 60±5% relative humidity, under a 12/12 hr natural light/dark cycle for a short duration. The final instar larvae were dissected to remove the gut and were subsequently ground and centrifuged (5,000 rpm, 10 min, 4^o^C) to separate the nuclei and remove the cell debris from the solution.

### Chemicals

All chemicals used were of analytical grade, and were obtained from Sigma Chemical Co. (St. Louis, MO, USA) until indicated otherwise.

### Preparation of High Molecular Weight (HMW) DNA Plugs Embedded in Agarose

HMW DNA is considered vulnerable to mechanical shearing forces and suffers frequent double-stranded breaks. It is thus not suited to large-insert cloning. To prevent HMW DNA from being damaged in the nucleus lysis process, the separated nuclei were embedded in agarose gel. The nuclei were warmed for 5 min at 45^o^C and were mixed with 1% InCert agarose. The mixture was subsequently poured into a plug mold (BioRad, Hercules, CA), kept on ice and allowed to solidify for 1–2 hr. The agarose plugs were then put into 50 ml of proteinase K lysis buffer (0.5 M EDTA, 1% N-lauroylascosine, 1 mg of proteinase K/ml) and incubated for 24 hr at 50^o^C. After the subsequent removal of proteinase K lysis buffer from the agarose plugs, the lysis process was repeated, for a further 24 hr. After 2–3 washes in deionized water, the plugs were placed in 50 ml of TE_50_ buffer (10 mM Tris-HCl, 50 mM EDTA, pH 8.0) and washed for 12 hr. Additional washing was performed for another 12 hr after replacing with TE_50_ buffer. Subsequently, the plugs were incubated for 2 hr in 0.1 mM phenylmethylsulfonylfluoride (PMSF) buffer at 4^o^C to inactivate proteinase K, followed by another subsequent wash in TE_50_ buffer for 24 hr, and were stored in 0.5 M EDTA at 4^o^C.

### Pre-electrophoresis of Agarose Plugs

Next, the agarose plugs were placed in 0.5× TBE buffer (45 mM Tris-base, 1 mM EDTA, 45 mM boric acid) and dialyzed for 3 hr. Subsequently, they were inserted into the preparative slot of 1% pulse- field certified agarose gel, and PFGE was conducted using 0.5× TBE buffer and the CHEF DR-II apparatus (Bio-Rad, Hercules, CA) with a pulse time of 5 s for 10 hr at 12^o^C and a voltage of 4V/cm. After the electrophoresis, the plugs were removed from the slot, stored in 50 ml of 0.5 M EDTA buffer, and dialyzed overnight at 4^o^C.

### Partial Digestion of Plugs

HMW DNA embedded plugs (*n = 10*) were placed in 500 µl of an enzyme mixture, consisting of 1 µl *Eco*RI at a concentration of 2 U/µl, 1 µl *Eco*RI methylase at a concentration of 40 U/µl (New England Biolabs, Ipswich, MA), 25 µl of 100× Bovine Serum Albumin (10 mg/ml), 5 µl of polyamine (100×), 50 µl of methylase buffer (10×) in 394 µl of DW and equilibrated for 2 hr at 4^o^C, followed by a 4 hr incubation at 37^o^C. After digestion, the plugs were treated with 150 µl of 0.5 M EDTA, 37.5 µl of 20% N-lauroylsarcosine and 15 µl of proteinase K (20 mg/ml), and incubated for 1 hr at 37^o^C to inactivate the endonuclease. Subsequently, PFGE was conducted with a CHEF DR-II apparatus (Bio-Rad) with a pulse time between 0.1 and 40 s for 16 hr at a voltage of 6 V/cm to check the partially digested plugs.

### Separation of PiraGV-K DNA from *P. rapae* Genomic DNA

PiraGV-K DNA was separated by PFGE with an initial pulse time of 0.1 s, a final pulse time of 40 s, a temperature of 12^o^C and a voltage of 6 V/cm for 14 hr. Furthermore, a lambda ladder PFG marker (New England Biolabs, Ipswich, MA) was used as a size marker to enable the band of PiraGV-K at ∼125 kb to be eluted selectively.

After the PFGE treatment, the edge of the gel, including a size marker, was cut and put into ethidium bromide staining buffer to mark the location of the 125 kb band of PiraGV-K. Subsequently, the eluted portion was placed into a dialysis bag to recover the PiraGV-K DNA using PFGE with a pulse time between 0.1 and 40 s and a voltage of 6 V/cm for 14 hr.

### Construction and Characterization of PiraGV-K Fosmid Library

Randomly sheared PiraGV-K DNA was cloned into the *Eco*72I blunt-end site of the CopyControl pCC1FOS fosmid vector (Epicentre Biotechnologies, Madison, WI). The fosmids were packaged using ultra-high efficiency MaxPlax lambda packaging extracts and plated on TransforMax EPI300 *E. coli* (Epicentre Biotechnologies, Madison, WI). The quality of the constructed fosmid library was assessed using standard techniques. Of a total of 6,000 clones, 96 were picked randomly and the fosmids were end sequenced from both directions using the primers (forward sequencing primer 5′– GGATGTGCTGCAAGGCGATTAAGTTGG –3′ and reverse sequencing primer 5′– CTCGTATGTTGTGGAATTGTGAGC –3′) to the vector. Stand-alone BLAST was performed for the nucleotide sequences against a locally curated viral sequence database (http://edunabi.com/~prgv/).

### Whole Genome Shotgun Sequencing

Based on the mapping data in the locally curated viral sequence database (http://edunabi.com/~prgv/), a minimum tiling path was prepared and four fosmid library clones were selected to construct a shotgun library. The selected fosmid clones were named as NB-FOS-1-1-F40_A05A02 (27 kb), NB-FOS-1-1-F40_A23B06 (33 kb), NB-FOS-1-1-F40_C07D02 (32 kb) and NB-FOS-1-1-F40_E13E04 (37 kb). Equivalent volumes of fosmid DNA clones were digested with *Not*I to obtain 3-7 kb DNA pieces that were then ligated into a purified pUC118 *Bam*HI/BAP ready vector (Takara Bio Inc., Shiga, Japan) [13]. Ligated products were transformed into *E. coli* DH5α cells by electroporation and spread on LB (ampicillin, 100 µg/ml) plates. The quality of the library was checked for *E. coli* genomic DNA contamination and empty vector contamination by cross-match. Plasmid clones that were eight times larger than each of the selected clones were randomly picked for plasmid preparation and sequencing with M13 forward and reverse universal primers using an Applied Biosystems 3730 XL DNA analyzer (Applied Biosystems, Carlsbad, CA) using the cycle sequencing method with fluorescent dye terminators and AmpliTaq DNA polymerase (ABI PRISM BigDye Terminator Cycle Sequencing Ready Reaction, Perkin Elmer, Waltham, MA). Applied Biosystems sequencing software was used for lane tracking, trace extraction and data were transferred to UNIX workstations for further processing.

### Genomic DNA Assembly

Contigs were prepared using the software Pregap4, including PHRED [14], [15], PHRAP (www.phrap.org), and vector masking on the average read length, clustering and assembling a repeated sequence. The primer walking procedure was used to close remaining gaps. The map of the first clone selected from PiraGV-K was constructed and a clone capable of covering 60 k to 85 k was also screened.

### Sequence Analysis

Putative coding regions of PiraGV-K genome was predicted using the Genemark [16]; Glimmer [17] and AMIgene [18] open reading frame (ORF) finding software. ORFs of more than 150 bp were designated as putative genes; the overlap between any two ORFs was set to a maximum of 25 amino acids (aa); otherwise, the longer one was selected. Gene annotations and comparison of the sequences with those in public databases were carried out using the BLAST at National Centre for Biotechnology Information (NCBI) (http://www.ncbi.nlm.nih.gov/BLAST/). Multiple sequence analysis was performed using Clustal X and GeneDoc (2.7.0). The PiraGV-K genomic DNA sequence was deposited in GenBank under the accession number JX968491.

Twelve betabaculovirus genomes were used to identify gene conservation in PiraGV-K. These genomes were from *Adoxophyes orana* GV (AdorGV; NC_005038) [19], *Agrotis segetum* GV (AgseGV; NC_005839), *Choristoneura occidentalis* GV (ChocGV; NC_008168) [20], *Cryptophlebia leucotreta* GV (CrleGV; NC_005068) [7], *Cydia pomonella* GV (CypoGV; NC_002816) [21], *Helicoverpa armigera* GV (HearGV; NC_010240) [22], *Phthorimaea operculella* GV (PhopGV; NC_004062), *P. rapae* GV-Chinese isolate (PiraGV-C; NC_013797), *Plutella xylostella* GV (PlxyGV; NC_002593 [4], *Xestia c-nigrum* GV (XecnGV; NC_002331) [5], *Pseudaletia unipuncta* GV (PsunGV; NC_013772) and *Spodoptera litura* GV (SpliGV; NC_009503) [23]. Detailed descriptions of the putative PiraGV-K ORFs, including their positions in the genome, length, and their relationship with AdorGV, AgseGV, ChocGV, CrleGV, CypoGV, HearGV, PhopGV, PiraGV-C, PlxyGV, PsunGV, SpliGV, and XecnGV are presented in Table S1.

### Data Access

The whole-genome data of PiraGV-K and relevant sequence information has been maintained in a database at ‘http://edunabi.com/~prgv/’ for ready reference. The PiraGV-K whole genome sequence is registered under GenBank accession number JX968491.

## Results and Discussion

### The Electrophoretic Separation Method for PiraGV-K Whole-genome Sequencing

Today, most genome sequencing projects rely on the whole-genome shotgun (WGS) method, which uses the Sanger technique to sequence genomic libraries over conventionally mapped clones using bacterial artificial chromosome (BAC), cosmid or fosmid libraries [24]–[26]. Although WGS strategy has provided rapid access to new gene models from diverse organisms with continued improvements in the assemblers, read lengths and mate pair technologies, the resulting assemblies still remain highly fragmented with an incomplete genomic representation [27], [28]. This has helped the focus on BAC-based physical map construction and its integration with high-density genetic maps that have benefited from next-generation sequencing (NGS) platforms and high-throughput array platforms [29], [30]. In this context, fosmids, with a narrower insert range (average of 40 kb), stable maintenance, and easy production, have found wide applications in studies related to structural variation and the organization of genomes [30]–[32].

The selection of target substances from the environment is the most critical component for the implementation of suitable approaches for whole-genome sequencing. In the case of infectious viruses, the study of the genome is more cumbersome because these agents are difficult to culture and purify. Conventional methods for the purification of genomic DNA fragments present the drawback of obtaining a large number of populations from multiple locations to acquire sufficient high-quality DNA samples for sequence analysis.

**Figure 1:**
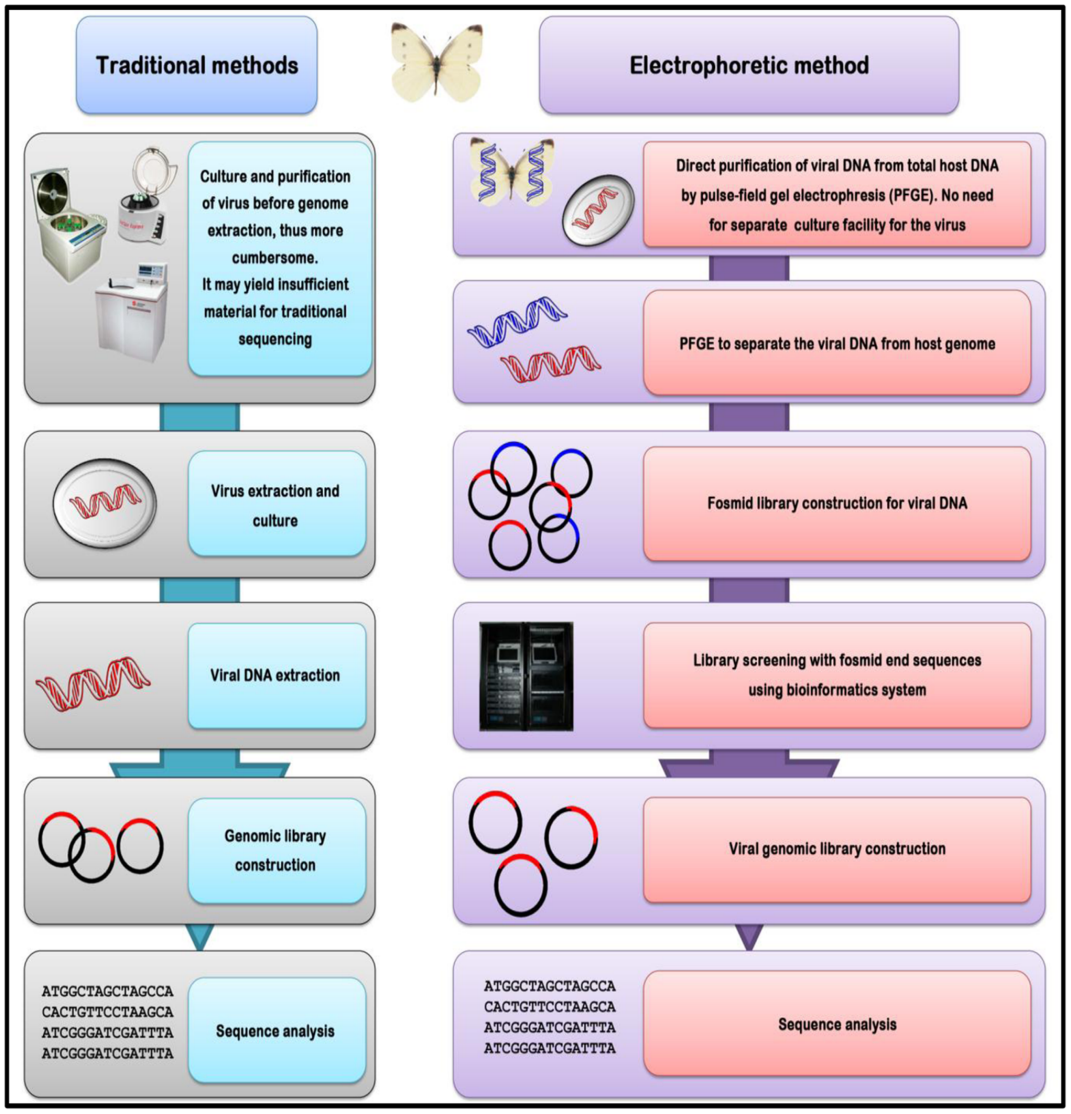
Comparative depiction of the electrophoretic and traditional methods for separation of viral genomic DNA. Flow chart showing the electrophoretic method for purification of the virus from the host genome for the construction of fosmid library of PiraGV-K and its significance in comparison with the traditional methods.

The genome sequencing method (Fig. 1), detailed here for the first time, was used to construct fosmid library clones of double-stranded PiraGV-K genome, generating a library size of 100–150 kb corresponding to the genome size of the virus. This approach was successful in the analysis of the PiraGV-K genome, without the need for purifying PiraGV-K from *P. rapae*, thus simplifying sampling and reducing labor time. This approach provides a significant advantage over traditional protocols for the sequencing of dsDNA genomes and could potentially be used for circular DNA genomes of viruses, although its wider application needs to be further validated. Recently, a report highlighted the importance of sequencing small genomes without the need for standard library preparation using the Pacific Biosciences RS sequencer (the “PacBio”) with as little as 1 ng of DNA [33]. That our method can be performed without the specialized expertise required for virus culturing and purification from their hosts, coupled with its requirement for little time and reliable precision, makes it particularly useful for laboratories lacking sophisticated viral culturing facilities. The limitations of the genome sequencing method purified by the electrophoretic method may lie in the sequencing of RNA viruses, because they are less stable than DNA in nature and may require the maintenance of cultured viral isolates, unlike our approach. A new system for rapid determination of viral RNA sequence (RDV) uses small amounts of RNA to synthesize first- and second- strand cDNAs for library construction and direct sequencing using optimized primers [34]. Although reverse transcription followed by polymerase chain reaction is commonly used for deciphering RNA viral genomes, low-copy number viral samples remain a challenge; sequence-independent methods provide attractive solutions [35], [36].

**Figure 2:**
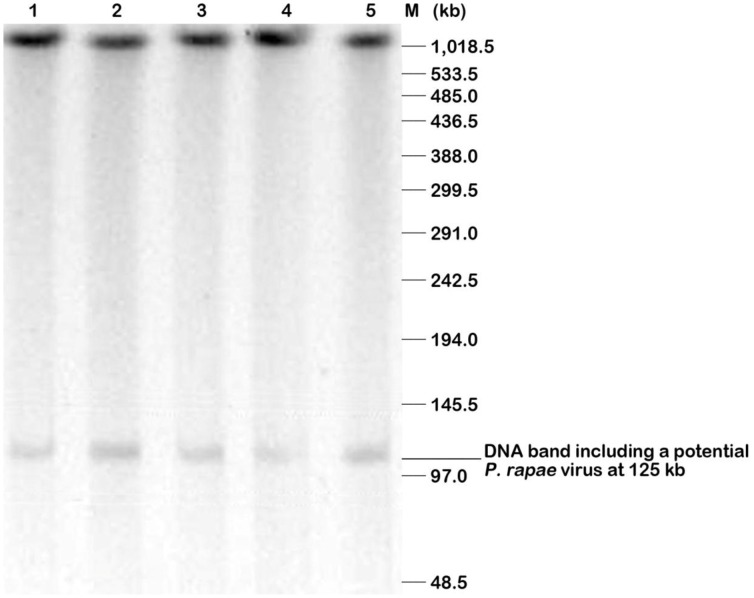
Pulse-field gel electrophoresis (PFGE) confirmation of the potential PiraGV-K DNA. HMW DNA embedded agarose plugs of *P. rapae* confirmed by PFGE, wherein the plugs were partially digested by an enzyme mixture following pre-electrophoresis. ‘M’ represents PFG lambda marker (NEB) and lanes 1–5 depict *Eco*RI digested DNA molds. A potential PiraGV-K DNA band was seen approximately at 125 kb after PFGE of enzyme digested DNA. PFGE conditions included 1% pulsed field certified agarose gel, a pulse time between 0.1–40 sec for up to 16 hrs and a voltage of 6 V/cm to check for partially digested plugs.

**Figure 3:**
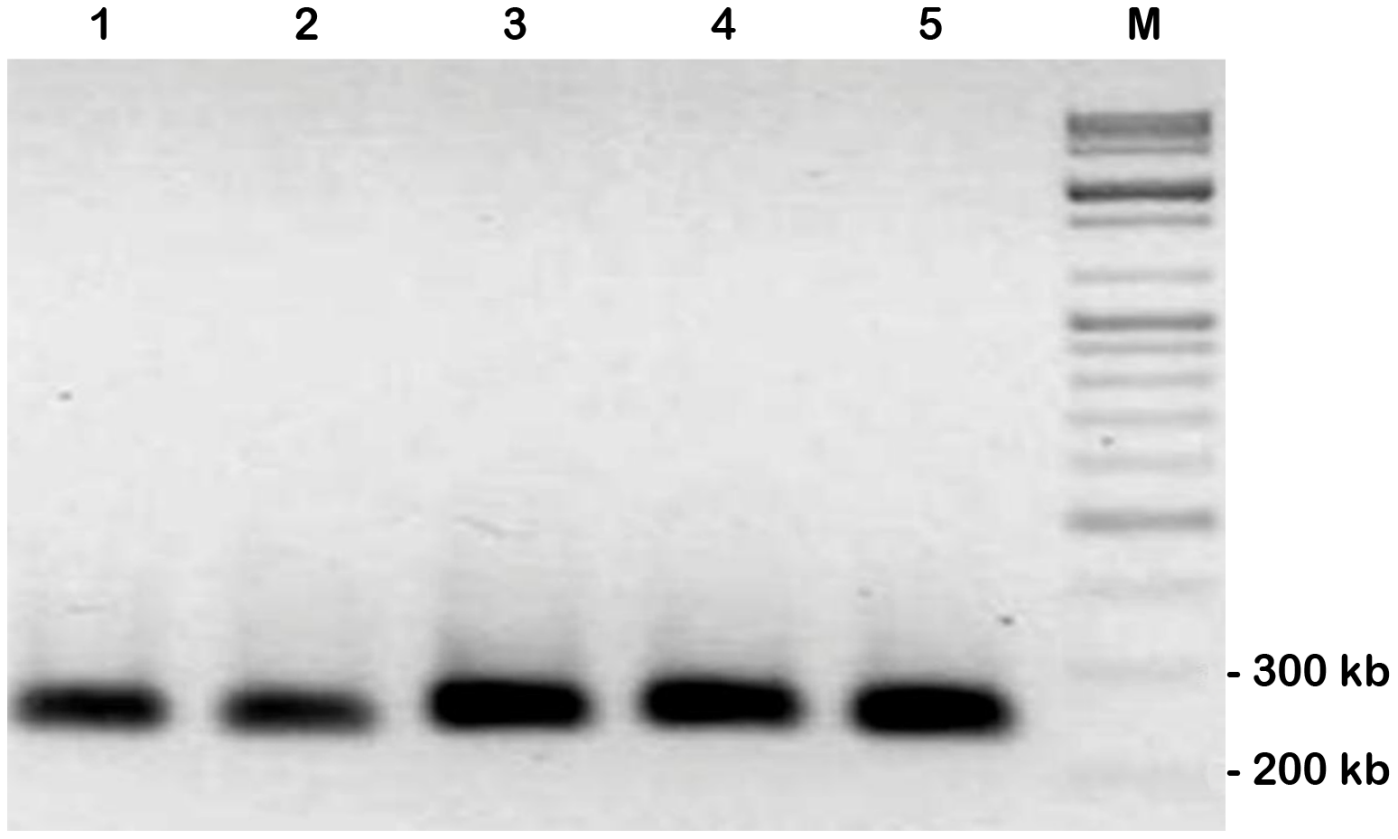
Confirmation of PiraGV-K DNA separated by PFGE. PCR was conducted to check the identity of PiraGV-K with 5 primers designed from the nucleotide sequence of PiraGV-K. The size of the PCR product was the same as the expected size of the nucleotide sequence. Lane 1; primer 1: AY-519253-1 (expected size of 227 bp), lane 2; primer 2: AY-706575-1 (expected size of 223 bp), lane 3; primer 3: AY-428513-1 (expected size of 234 bp), lane 4; primer 4: AY-449794-2 (expected size of 212 bp), lane 5; primer 5: AY-519252-1 (expected size of 231 bp).

**Figure 4:**
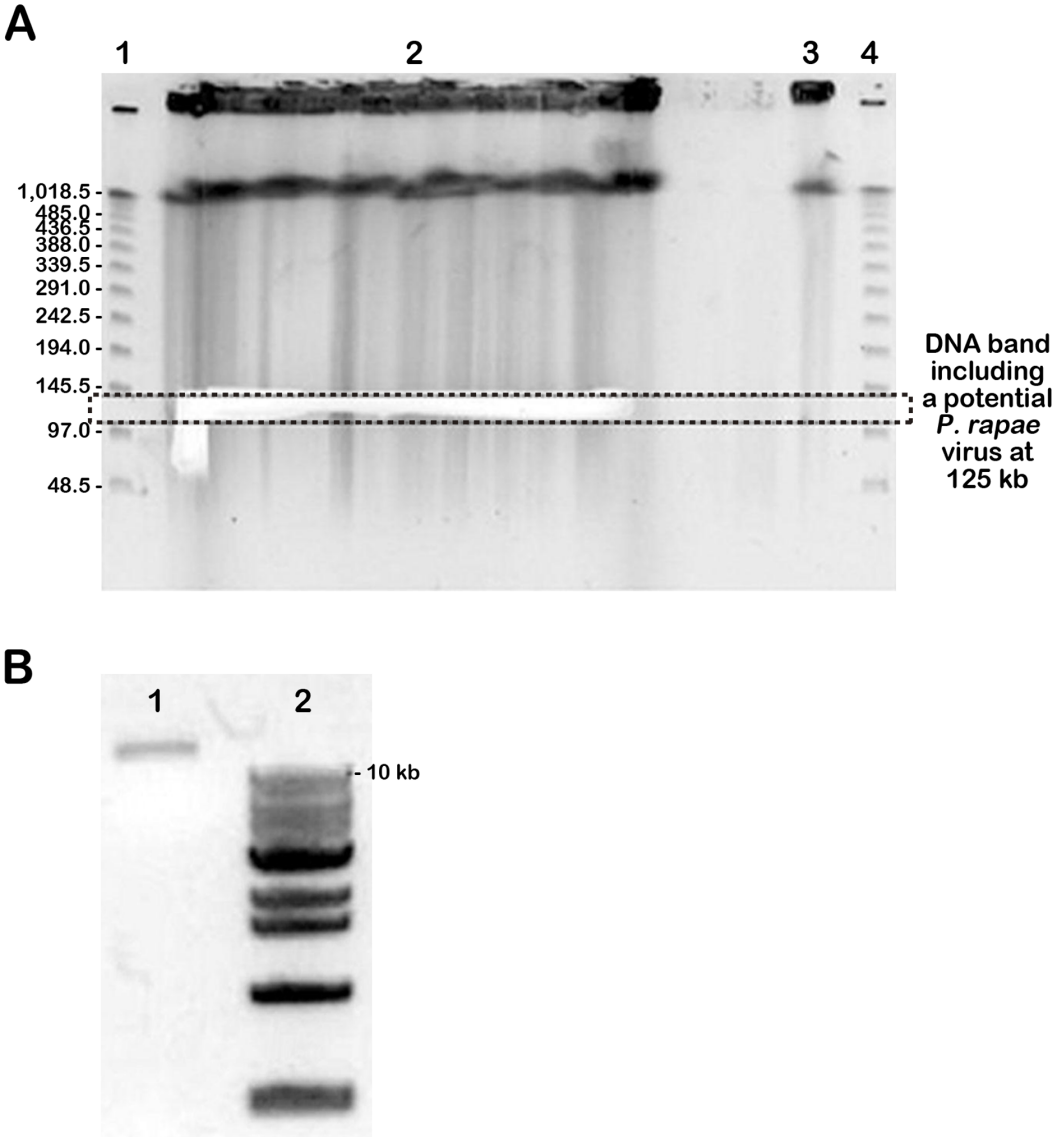
Separation of potential PiraGV-K DNA from agarose molds after PFGE. (A) Elution of DNA band (approximately 125 kb) of potential PiraGV-K. This indicates that the DNA of PiraGV-K is separated from *P. rapae* DNA embedded agarose molds. Lanes 1 and 4 show PFG lambda marker (NEB) and lanes 2 and 3 depict *Eco*RI digested DNA molds. (B) This indicates the concentration of DNA that has been collected by PFGE as determined using a spectrophotometer. Lanes 1 and 2 show eluted DNA (20 ng loading) and a 1 kb ladder, respectively.

**Figure 5:**
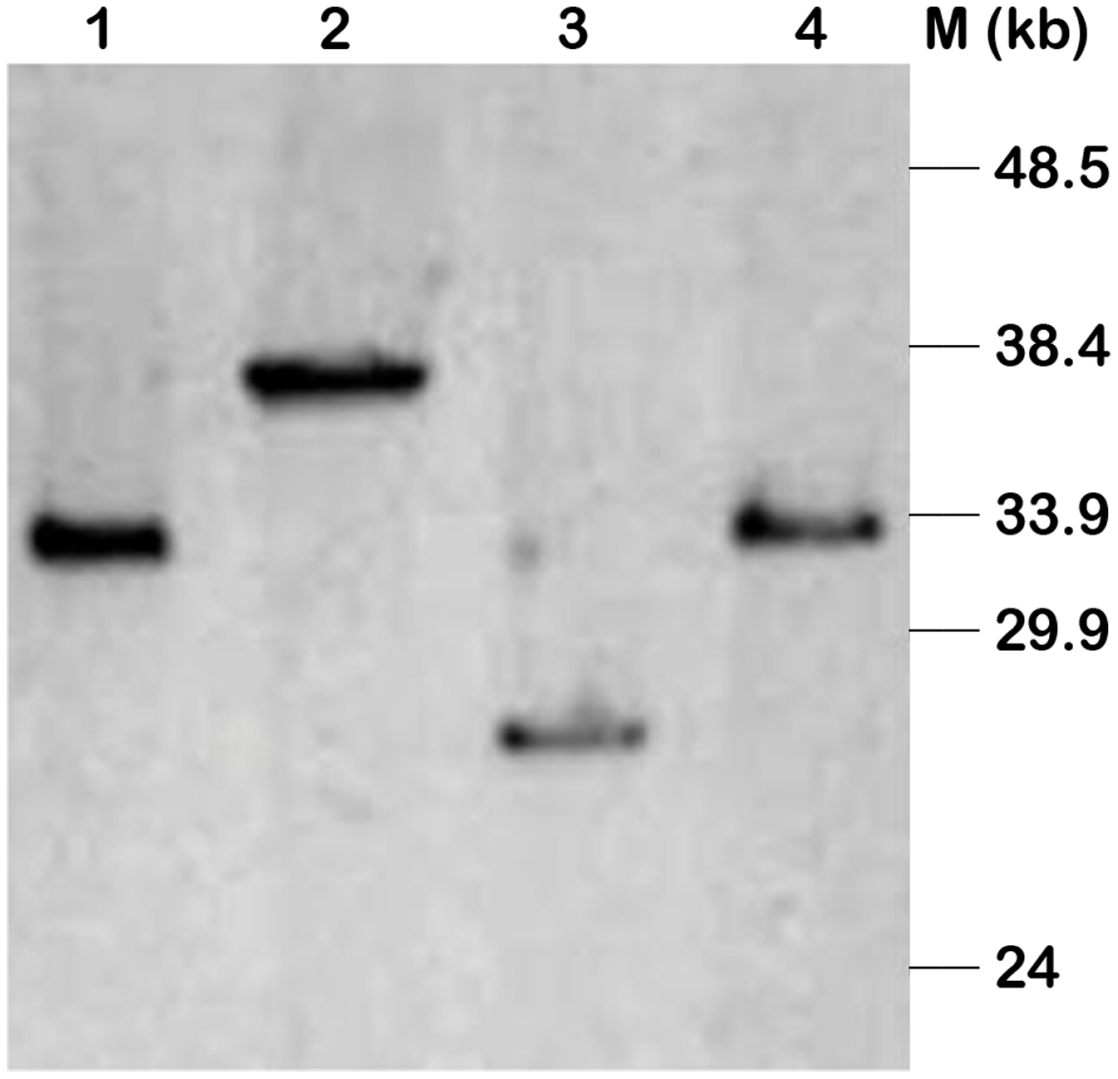
Restriction digestion of selected fosmid clone DNA by *Not*I enzyme. Four fosmid clones were selected on the basis of minimum tiling path towards construction of shotgun library. Lane 1, fosmid clone NB-FOS-1-1-F40_C07D02 (approximately 32 kb); Lane 2, fosmid clone NB-FOS-1-1-F40_E13E04 (approximately 37 kb); Lane 3, fosmid clone NB-FOS-1-1-F40_A05A02 (approximately 27 kb); Lane 4, fosmid clone NB-FOS-1-1-F40_A23B06 (approximately 33 kb). Lane ‘M’ is represented by monocot lambda marker.

**Figure 6:**
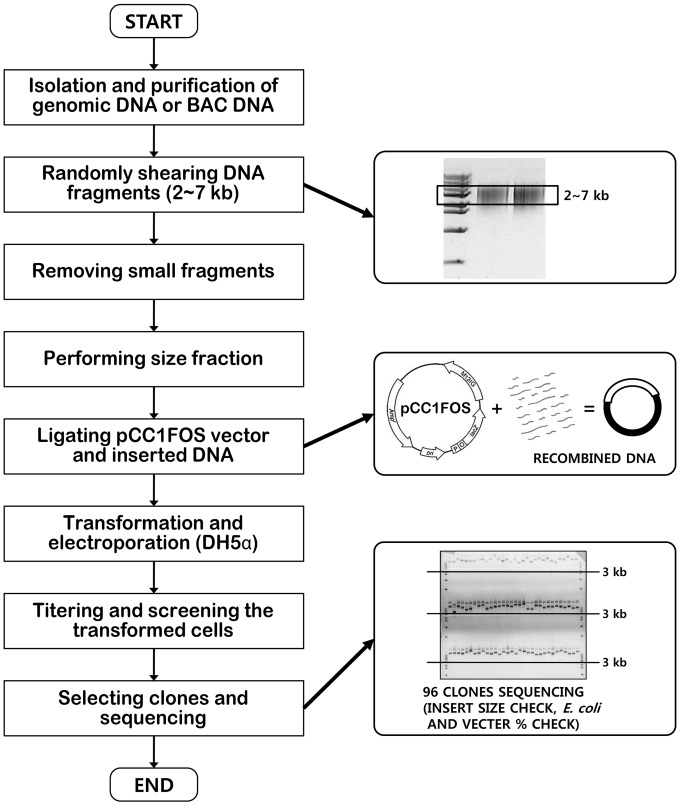
Flow chart depicting shotgun library construction. Genomic DNA or BAC DNA isolation and purification was followed by size fractionation and ligation into a pUC118 ready vector for 4^o^C followed with transformation by electroporation into DH5α. The quality of thus constructed shotgun library was checked by titering (40 µl of cell stock, white: blue = 400∶100). The number of clones was approximately 20,000 in total. 96 clones were selected and sequenced including insert size check, *E. coli* and vector % check.

In the method described here, HMW DNA embedded agarose plugs of *P. rapae* were digested with *Eco*RI, before confirmation of the potential PiraGV-K DNA at 125 kb by PFGE analysis (Fig. 2). The potential PiraGV-K DNA was found readily when *Eco*RI (8 U) and methylase (20 U) were used after a 2 hr pre-electrophoresis step. The partial digestion step is considered critical for both the construction of the host BAC library, and also converting the viral genome into a family of circularly-permuted linear molecules of genome length. The linear form of the viral genome, thus obtained from the digestion step facilitates efficient separation of the genomic DNA in PFGE. Subsequently, PCR was conducted with different primers, designed to provide variable sizes from the nucleotide sequence of PiraGV-K, to check the validity of the potential PiraGV-K DNA. The PCR product size in all cases was found to be the same as expected for the PiraGV-K DNA sequence (Fig. 3). Subsequently, for effective separation of PiraGV-K DNA, pre-electrophoresis and partial digestion of agarose plugs was repeated with PFGE. Following the PFGE run, the DNA band of 125 kb corresponding to PiraGV-K DNA was eluted, eventually separating PiraGV-K DNA from *P. rapae* embedded agarose molds (Fig. 4A). The eluted DNA (20 ng) was subsequently electrophoresed in parallel with a 1 kb ladder to validate the separation process (Fig. 4B). The eluted and end-repaired PiraGV-K DNA was ligated into the pCC1FOS vector and the purified products were checked for quality by titering. In total, approximately 6,000 clones resulted, out of which 96 were selected and end-sequenced. To effectively map the fosmid-end sequences, we performed a stand-alone BLAST against a locally constructed viral sequence database. Based on the mapping data from the databases, a minimum tiling path (MTP) was prepared, leading to the selection of four fosmid library clones for the construction of a PiraGV-K shotgun library. The sizes of the four selected fosmid clones, (NB-FOS-1-1-F40_C07D02, NB-FOS-1-1-F40_E13E04, NB-FOS-1-1-F40_A05A02, and NB-FOS-1-1-F40_A23B06), measured by *Not*I restriction digestion were approximately 32, 37, 27, and 33 kb, respectively (Fig. 5). The shotgun library resulted in a total of 20,000 clones, of which 96 were selected and sequenced (Fig. 6).

### Characteristics of the PiraGV-K Genome Sequence

**Figure 7:**
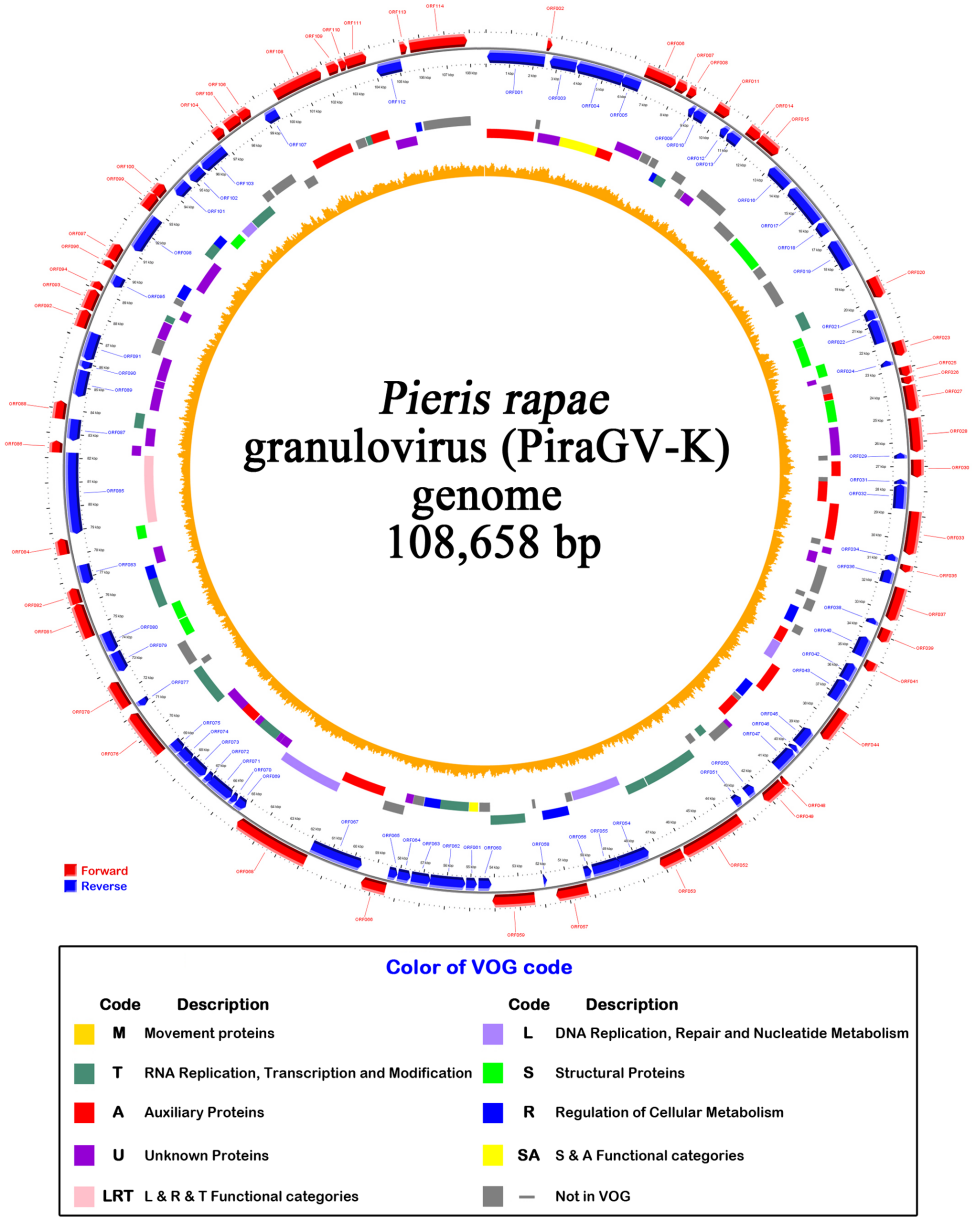
Circular representation of the PiraGV-K genome map. ORFs are represented by arrows, with the position and direction of the arrow indicating ORF position and orientation. Red arrows and blue arrows represent forward and reverse strand ORFs in the circular map. VOG code and colors assigned indicate the grouping of the genes according to function.

To date, whole-genome sequencing has been conducted successfully for 60 baculoviruses: 45 were NPVs (41 alphabaculoviruses, 3 gammabaculoviruses and 1 deltabaculovirus). Only 14 complete genomes have been sequenced of betabaculoviruses, including PiraGV-C [12]. The growing number of fully sequenced baculovirus genomes now allows some understanding of the evolutionary history of baculoviruses by comprehensive analyses of nucleotide/protein sequences, gene order, and content [37], [38]. We have sequenced and analyzed the 108,658 bp PiraGV-K genome purified by electrophoretic method. The approach allows for the determination of the viral sequence with multiple fold redundancy per base position. An 8x sequence of the PiraGV-K genome was compiled from the sequence data generated here. The size of the final draft sequence was 108,658 nt (Fig. 7). The length of the sequence obtained was consistent with the predicted size of PiraGV-C (108,592 nt), differing by only 66 nt. It can thus be categorized as one of the smaller betabaculoviruses sequences, with AdorGV (99,657 nt) being the smallest. XecnGV has a whole genome size of 178,733 nt [5], which is largest genome among the completely sequenced betabaculoviruses and is closely related to sequences studied from noctuid moths, including *Autographa gamma* GV, *Hoplodrina ambigua* GV, *Euxoa ochrogaster* GV, and *Scotogramma trifolii* GV [39]. These are closely followed by HearGV, with a genome size of 169,794 bp [40]. PiraGV-K coding sequences represent 92% of the genome, leaving very little noncoding DNA.

**Figure 8:**
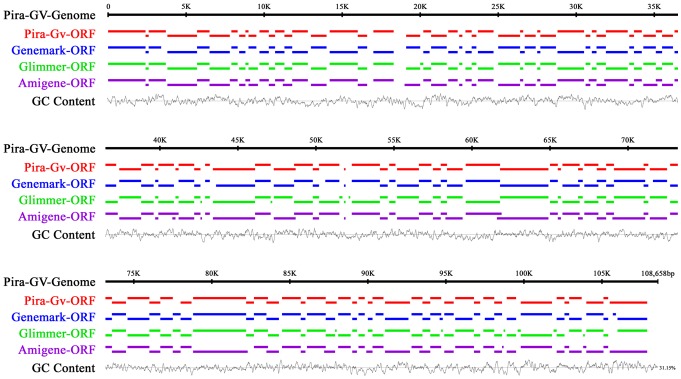
Predictive map of the putative coding regions of PiraGV-K genome. The putative coding regions were predicted using GeneMark (Georgia Institute of Technology, Atlanta, Georgia), Glimmer-Microbial gene-finding system (University of Maryland) and AMIgene-an integrated computer environment for sequence annotation and analysis (Institut Pasteur, France) ORFs finding softwares.

The PiraGV-K genome has an AT content of 66%, identical to PiraGV-C (66%), and is closely related to CrleGV, having the highest known AT content of 67.6%. This result is consistent with previous findings that the sequenced betabaculovirus genomes are AT-rich, with the lowest AT content of 54.8% observed in case of CypoGV, with an overall average of 62.6%. The difference in AT content is due to the base composition at the third nucleotide position of the codon in the coding regions. It has been established previously that proteins encoded by more extreme AT and GC-rich genomes generally have lower compositional complexity than those of more typical organisms [41]. A consequence of this is that the overall amino acid composition of the peptides in such organisms is skewed. Peptides of AT-rich organisms have higher proportions of Phe, Leu, Ile, Met, Asn, Lys and Tyr that are relatively rare in the organisms with GC-rich genomes. Similar correlation has been noted with smaller data sets in earlier research [19], [42], [43]. The end result of this is that organisms with an extreme genome composition encode peptides of a lower complexity, as measured by the global complexity value [44]. It is known that the median global complexity value, G1 for AT-rich genes from a variety of cellular organisms is in the range of 0.72 to 0.78 [41]. Whereas most PiraGV-K ORFs had an AT composition (average 65%) close to the average AT composition of PiraGV-K genome (66%), *granulin* had an AT composition that was significantly lower at 56% (results not shown). It is to be noted that in case of extremely anchored proteins, such as *granulin*, it might be impossible for the virus to maintain its preferred nucleotide composition and codon usage and still encode a particular peptide. This observation has been confirmed in other annotated, AT-rich, viral genomes [19], [45] Also, it is understood that, although various ORF prediction methods have been used (Fig. 8), no one method can define all possible ORFs in compositionally extreme (AT or GC-rich) genomes, as is clearly illustrated in the PiraGV-K genome. PiraGV-K *granulin* had a subjective appearance of an “alien” gene, because the codon usage did not conform to the overall codon usage [46]. However, we believe that *granulin* represents a specific class of highly expressed, complex peptide that the virus encodes by sacrificing the constraints it maintains on other genes.

The primary criteria used to identify potential ORFs on the PiraGV-K genome were a minimum of 50 aa in length, having minimal overlap with larger ORFs, and sharing significant sequence identity with previously characterized ORFs of betabaculoviruses (Table 1). Also, by convention, the first nucleotide of the methionine start codon of *granulin* was defined as the first nucleotide of the genome, and the sequence was numbered in the direction of transcription of the gene. As in the case of other baculovirus genomes, minimal overlaps were observed in the PiraGV-K genome sequence with 65 ORFs in the *granulin*-sense orientation and, 54 in the opposite orientation, clustering together according to expression or function. Homologous repeat regions (*hrs*), functioning as enhancers of transcription and origins of replication, were also found interspersed in the genome. These repeated sequences have been reported to be more variable in betabaculoviruses than in alphabaculoviruses, where they consist of repeated palindromes. The CypoGV genome includes 13 *hrs,* as do the XecnGV and HearGV genomes. The AdorGV genome includes nine repeated regions that are unlike typical *hrs* [19]. Six repeat regions, including one unique *hrs*, have also been identified in the EppoMNPV genome [47]. In the completely sequenced genome of SpltNPV, 17 *hrs* were identified [48]. In the AcMNPV, *hrs* consist of repeated units of about 70 bp with an imperfect 30 bp palindrome near their center, binding to the transcriptional activator *ie1* (Ac147) [49]. Also, cAMP and 12-*O*-tetradecanoylphorbol 13-acetate (TPA) response elements (CRE and TRE)-like sequences, located between *hrs* palindromes have been found to be evolutionarily conserved in alphabaculoviruses, but were not found in betabaculoviruses.

**Table 1:** Analysis and annotation of PiraGV-K ORFs.

QueryID	SubjectID	Annotation	Pid	Psi	Eval	Db	Best Hit Annotation	Source
GVORF001	AAR06236.1	granulin	100	100	0	gb	granulin	*P. rapae* granulovirus-Chinese strain
GVORF002	YP_654423.1	hypothetical protein COGV_gp002	61.9	73	6E-20	ref	hypothetical protein COGV_gp002	*C. occidentalis* granulovirus
GVORF003	YP_654424.1	pk-1	67	83.3	1E-141	ref	pk-1	*C. occidentalis* granulovirus
GVORF004	NP_148788.1	ORF4 similar to *Cydia pomonella* GV ORF4	65.4	81.4	7E-92	ref	ORF4 similar to XcGV ORF7	*C. pomonella* granulovirus
GVORF005	YP_654427.1	hypothetical protein COGV_gp006	60.3	74.4	1E-25	ref	hypothetical protein COGV_gp006	*C. occidentalis* granulovirus
GVORF006	YP_654428.1	ie-1	52.3	72.6	1E-144	ref	ie-1	*C. occidentalis* granulovirus
GVORF007	YP_654429.1	hypothetical protein COGV_gp008	58.1	75.3	1E-77	ref	hypothetical protein COGV_gp008	*C. occidentalis* granulovirus
GVORF008	NP_148793.1	ORF8 similar to *Cydia pomonella* GV ORF9	69.3	88.1	1E-55	ref	ORF9 similar to AcMNPV ORF145	*C. pomonella* granulovirus
GVORF009		unknown					No hit	No hit
GVORF010	NP_148794.1	chitinase	65.3	78.2	0	ref	ORF10 chitinase	*C. pomonella* granulovirus
GVORF011	NP_148795.1	cathepsin	69.2	82.8	1E-180	ref	ORF11 cathepsin	*C. pomonella* granulovirus
GVORF012	NP_148796.1	unknown	60.4	77.4	1E-18	ref	ORF12	*C. pomonella* granulovirus
GVORF013	NP_148797.1	gp37	59.8	75.2	1E-111	ref	ORF13 gp37	*C. pomonella* granulovirus
GVORF014	YP_001256961.1	odv-e18	62.2	73	2E-21	ref	odv-e18	*S. litura* granulovirus
GVORF015	YP_654434.1	p49	63.8	80	0	ref	p49	*C. occidentalis* granulovirus
GVORF016	YP_654435.1	odv-e56	72.1	85.8	0	ref	odv-e56	*C. occidentalis* granulovirus
GVORF017	NP_068221.1	ORF17 similar to PxORF2 peptide	37	51	6E-13	ref	PxORF2 peptide	*P. xylostella* granulovirus
GVORF018	NP_872468.1	ORF18 similar to *Adoxophyes orana* granulovirus ORF14	31.8	48.5	7E-12	ref	ORF_14	*A. orana* granulovirus
GVORF019	NP_148803.1	ORF19 similar to *Cydia pomonella* GV ORF19	48.7	65.4	1E-18	ref	ORF19 similar to AcMNPV ORF29	*C. pomonella* granulovirus
GVORF020	NP_148804.1	pep1	51.7	63.7	2E-74	ref	ORF20 similar to XcGV ORF17	*C. pomonella* granulovirus
GVORF021	NP_148806.1	calyx/polyhedral envelope protein	67.9	78.3	1E-170	ref	ORF22 similar to XcGV ORF19	*C. pomonella* granulovirus
GVORF022	NP_148807.1	pep2	66.7	76.4	3E-60	ref	ORF23 similar to XcGV ORF18	*C. pomonella* granulovirus
GVORF023	NP_148808.1	pe-38	28.9	47.8	1E-14	ref	ORF24 PE-38	*C. pomonella* granulovirus
GVORF024	YP_654443.1	hypothetical protein COGV_gp022	29.2	44	5E-32	ref	hypothetical protein COGV_gp022	*C. occidentalis* granulovirus
GVORF025	NP_148814.1	ORF25 similar to *Cydia pomonella* GV ORF30	41.5	63.6	7E-41	ref	ORF30 similar to XcGV ORF26	*C. pomonella* granulovirus
GVORF026	NP_148815.1	ORF26 similar to *Cydia pomonella* GV ORF31	64.1	80.4	0	ref	ORF31 similar to AcMNPV ORF23	*C. pomonella* granulovirus
GVORF027	ZP_01774245.1	ORF27 similar to *Adoxophyes orana* GV ORF24	33	40.9	1E-20	ref	conserved hypothetical protein	*G. bemidjiensis* Bem
GVORF028	NP_148817.1	ORF28 similar to *Cydia pomonella* GV ORF33	37.5	55.1	1E-58	ref	ORF33 similar to XcGV ORF29	*C. pomonella* granulovirus
GVORF029	NP_891880.1	unknown	63	77.2	8E-83	ref	hypothetical protein ClgVgp033	*C. leucotreta* granulovirus
GVORF030	NP_148819.1	ORF30 similar to *Cydia pomonella* GV ORF35	58.5	76.1	1E-83	ref	ORF35 similar to AcMNPV ORF115	*C. pomonella* granulovirus
GVORF031	NP_148823.1	ORF31 similar to *Cydia pomonella* GV ORF39	72.5	90.2	6E-55	ref	ORF39 similar to XcGV ORF34	*C. pomonella* granulovirus
GVORF032	YP_003429356.1	phosphohydrolase	99	100	1E-141	ref	phosphohydrolase	*P. rapae* granulovirus-Chinese strain
GVORF033	YP_654450.1	lef-2	61.3	78	3E-72	ref	lef-2	*C. occidentalis* granulovirus
GVORF034	NP_148826.1	hypothetical protein COGV_gp030	48.8	65.9	4E-20	ref	ORF42 similar to XcGV ORF36	*C. pomonella* granulovirus
GVORF035	YP_654452.1	hypothetical protein COGV_gp031	45.9	67.6	3E-29	ref	hypothetical protein COGV_gp031	C. *occidentalis* granulovirus
GVORF036	YP_654453.1	hypothetical protein COGV_gp032	55	73.8	3E-40	ref	hypothetical protein COGV_gp032	*C. occidentalis* granulovirus
GVORF037	NP_891890.1	metalloproteinase	46.6	64.9	1E-137	ref	metalloproteinase	*C. leucotreta* granulovirus
GVORF038	NP_148831.1	p13	59.7	75.4	1E-108	ref	ORF47 p13	*C. pomonella* granulovirus
GVORF039	NP_148821.1	odv-e66	31.2	50.8	1E-59	ref	ORF37 odv-e66	*C. pomonella* granulovirus
GVORF040	YP_654456.1	pif-2	71.1	83.5	0	ref	pif-2	*C. occidentalis* granulovirus
**QueryID**	**SubjectID**	**Annotation**	**Pid**	**Psi**	**Eval**	**Db**	**Best Hit Annotation**	**Source**
GVORF041	NP_663210.1	hypothetical protein PogVgp045	46.9	73.4	8E-17	ref	hypothetical protein PogVgp045	*P. operculella* granulovirus
GVORF042	NP_148834.1	ORF42 similar to *Cydia pomonella* GV ORF050	41.4	59.4	2E-51	ref	ORF50 similar to XcGV ORF47	*C. pomonella* granulovirus
GVORF043	NP_891897.1	ORF43 similar to *Cryptophlebia leucotreta* GV ORF50	78	88	1E-119	ref	hypothetical protein ClgVgp050	*C. leucotreta* granulovirus
GVORF044	NP_047452.1	odv-e66	70.1	83.2	0	ref	odv-e66	*B. mori* NPV
GVORF045	YP_654460.1	UBQ	89.2	93.5	6E-59	ref	UBQ	*C. occidentalis* granulovirus
GVORF046	YP_654461.1	hypothetical protein COGV_gp040	65.8	80.8	1E-176	ref	hypothetical protein COGV_gp040	*C. occidentalis* granulovirus
GVORF047	NP_148840.1	ORF47 similar to *Cydia pomonella* GV ORF56	53.1	76.6	5E-21	ref	ORF56 similar to XcGV ORF54	*C. pomonella* granulovirus
GVORF048	YP_654463.1	39K	48.7	67	1E-75	ref	39K	*C. occidentalis* granulovirus
GVORF049	YP_654464.1	lef-11	65.2	79.3	5E-40	ref	lef-11	*C. occidentalis* granulovirus
GVORF050	NP_663219.1	superoxide dismutase	67.3	82.7	3E-78	ref	superoxide dismutase	*P. operculella* granulovirus
GVORF051	NP_148844.1	ORF51 similar to p74 (Baculoviridae p74 conserved region	61.7	76.2	0	ref	ORF60 p74	*C. pomonella* granulovirus
GVORF052	YP_610994.1	p22.2	34.3	49.7	8E-29	ref	p22.2	*A. pernyi* nucleopolyhedrovirus
GVORF053	YP_743425.1	dehydrogenase catalytic domain-containing protein	71.7	77.4	3E-12	ref	dehydrogenase catalytic protein	*A. ehrlichei* MLHE-1
GVORF054	YP_654469.1	hypothetical protein COGV_gp048	61.6	77.8	4E-88	ref	hypothetical protein COGV_gp048	*C. occidentalis* granulovirus
GVORF055	NP_891907.1	ORF55 similar to *Cryptophlebia leucotreta* GV ORF60	71.4	88.9	7E-26	ref	hypothetical protein ClgVgp060	*C. leucotreta* granulovirus
GVORF056	YP_654471.1	p47	71.1	83.2	0	ref	p47	*C. occidentalis* granulovirus
GVORF057	NP_891909.1	Nudix_Hydrolase	77.8	90.5	1E-129	ref	hypothetical protein ClgVgp062	*C. leucotreta* granulovirus
GVORF058	YP_654473.1	p24 capsid protein	60	79.4	5E-78	ref	p24 capsid protein	*C. occidentalis* granulovirus
GVORF059	YP_654475.1	38.7KD protein	44.5	63	3E-35	ref	38.7KD protein	*C. occidentalis* granulovirus
GVORF060	YP_654476.1	lef-1	68.7	82.4	1E-126	ref	lef-1	*C. occidentalis* granulovirus
GVORF061	YP_654477.1	pif-1	63.7	79.1	0	ref	pif-1	*C. occidentalis* granulovirus
GVORF062	YP_654478.1	fgf-1	58.9	77.2	6E-98	ref	fgf-1	*C. occidentalis* granulovirus
GVORF063	YP_654479.1	COGV_gp058 contains chitin binding domain	42	63	1E-18	ref	hypothetical protein COGV_gp058	*C. occidentalis* granulovirus
GVORF064	NP_872520.1	ORF64 similar to *Adoxophyes orana* GV ORF66	35.3	52.7	6E-28	ref	ORF_66	*A. orana* granulovirus
GVORF065	YP_654481.1	lef-6	52.5	72.5	4E-27	ref	lef-6	*C. occidentalis* granulovirus
GVORF066	YP_654482.1	DBP	66.8	82.8	1E-136	ref	DBP	*C. occidentalis* granulovirus
GVORF067	YP_654484.1	hypothetical protein COGV_gp063	35	52.9	2E-34	ref	hypothetical protein COGV_gp063	*C. occidentalis* granulovirus
GVORF068	NP_148867.1	ORF68 similar to *Cydia pomonella* GV ORF83	80.9	90.7	0	ref	ORF83 similar to AcMNPV ORF103	*C. pomonella* granulovirus
GVORF069	NP_148868.1	ORF69 similar to *Cydia pomonella* GV ORF84	65.7	79.4	5E-43	ref	ORF84 similar to AcMNPV ORF102	*C. pomonella* granulovirus
GVORF070	YP_654487.1	hypothetical protein COGV_gp066	70.6	85.6	0	ref	hypothetical protein COGV_gp066	*C. occidentalis* granulovirus
GVORF071	YP_654488.1	p6.9	81	87.9	2E-17	ref	p6.9	*C. occidentalis* granulovirus
GVORF072	NP_148871.1	lef-5	71.8	83.7	1E-129	ref	ORF87 lef-5	*C. pomonella* granulovirus
GVORF073	YP_654490.1	hypothetical protein COGV_gp069	70.8	84.2	1E-161	ref	hypothetical protein COGV_gp069	*C. occidentalis* granulovirus
GVORF074	NP_148873.1	19KD	67.5	81.9	4E-72	ref	ORF89 similar to AcMNPV ORF96	*C. pomonella* granulovirus
GVORF075	YP_654492.1	helicase-1	64.5	80	0	ref	helicase-1	*C. occidentalis* granulovirus
GVORF076	NP_148875.1	odv-e25	81.3	93.9	1E-131	ref	ORF91 odv-e25	*C. pomonella* granulovirus
GVORF077	NP_148876.1	ORF77 similar to *Cydia pomonella* GV ORF92	60.4	79.9	2E-66	ref	ORF92 similar to AcMNPV ORF93	*C. pomonella* granulovirus
GVORF078	NP_148877.1	ORF78 similar to *Cydia pomonella* GV ORF93	68.5	88	1E-142	ref	ORF93 similar to AcMNPV ORF92	*C. pomonella* granulovirus
GVORF079	NP_891932.1	iap	37	60.2	1E-48	ref	iap	*C. leucotreta* granulovirus
GVORF080	NP_148879.1	lef-4	56.9	73.5	0	ref	ORF95 lef-4	*C. pomonella* granulovirus
**QueryID**	**SubjectID**	**Annotation**	**Pid**	**Psi**	**Eval**	**Db**	**Best Hit Annotation**	**Source**
GVORF081	YP_654497.1	vp39 capsid	73.4	84.3	1E-158	ref	vp39 capsid	*C. occidentalis* granulovirus
GVORF082	YP_654498.1	odv-e27	71.4	86.4	1E-148	ref	odv-e27	*C. occidentalis* granulovirus
GVORF083	YP_654499.1	hypothetical protein COGV_gp078	51.9	71.1	1E-129	ref	hypothetical protein COGV_gp078	*C. occidentalis* granulovirus
GVORF084	NP_891938.1	ORF084 similar to *Cryptophlebia leucotreta* ORF91	59	73.8	2E-20	ref	hypothetical protein ClgVgp091	*C. leucotreta* granulovirus
GVORF085	NP_891939.1	vp91 capsid	46.5	67.2	0	ref	vp91 capsid	*C. leucotreta* granulovirus
GVORF086	NP_891940.1	tlp20	39	54.2	9E-25	ref	tlp20	*C. leucotreta* granulovirus
GVORF087	NP_891941.1	hypothetical protein ClgVgp094	79.2	94	1E-112	ref	hypothetical protein ClgVgp094	*C. leucotreta* granulovirus
GVORF088	NP_148888.1	Structural glycoprotein p40/gp41 conserved	73.8	88.1	1E-164	ref	ORF104 GP41	*C. pomonella* granulovirus
GVORF089	YP_654506.1	hypothetical protein COGV_gp085	51.6	64.2	6E-23	ref	hypothetical protein COGV_gp085	*C. occidentalis* granulovirus
GVORF090	NP_891944.1	vlf1 (very late expression factor 1)	73.8	87.4	0	ref	vlf-1	*C. leucotreta* granulovirus
GVORF091	NP_148891.1	ORF91 similar to *Cydia pomonella* GV ORF107	73.8	89.3	2E-43	ref	ORF107 similar to AcMNPV ORF76	*C. pomonella* granulovirus
GVORF092	ABC67291.1	unknown	100	100	1e-104,	gb	unknown	*P. rapae* granulovirus-Chinese strain
GVORF093	ACZ63579.1	DNA polymerase	99.7	99.9	0.0,	gb	DNA polymerase	*P. rapae* granulovirus-Chinese strain
GVORF094	ACZ63580.1	desmoplakin	100	100	0.0,	gb	desmoplakin	*P. rapae* granulovirus-Chinese strain
GVORF095	ACZ63581.1	lef-3	100	100	0.0,	gb	lef-3	*P. rapae* granulovirus-Chinese strain
GVORF096	ABC67295.1	unknown	100	100	6e-89,	gb	unknown	*P. rapae* granulovirus-Chinese strain
GVORF097	ABC67296.1	unknown	99.4	100	1e-123,	gb	unknown	*P. rapae* granulovirus-Chinese strain
GVORF098	ABC67297.1	iap-5	99.6	100	0.0,	gb	iap-5	*P. rapae* granulovirus
GVORF099	ABC67298.1	lef-9	100	100	0.0,	gb	lef-9	*P. rapae* granulovirus
GVORF100	ABC67299.1	fp	100	100	1e-110,	gb	fp	*P. rapae* granulovirus
GVORF101	ABC67300.1	unknown	100	100	1e-126,	gb	unknown	*P. rapae* granulovirus
GVORF102	NP_148904.1	DNA ligase	66.1	80.5	0	ref	ORF120 DNA LIGASE	*C. pomonella* granulovirus
GVORF103	YP_004376313.1	hypothetical protein ClanGV_gp105	55.3	68.4	0.000001	ref	hypothetical protein ClanGV_gp105	*C. anachoreta* granulovirus
GVORF104	YP_654522.1	hypothetical protein COGV_gp101	60.9	79.7	3E-22	ref	hypothetical protein COGV_gp101	*C. occidentalis* granulovirus
GVORF105	NP_891960.1	fgf-2	44.7	66.6	1E-106	ref	fgf	*C. leucotreta* granulovirus
GVORF106	NP_663280.1	ORF106 similar to *Phthorimaea operculella* GV ORF115	63	85.9	2E-42	ref	hypothetical protein PogVgp115	*P. operculella* granulovirus
GVORF107	YP_654525.1	ALK-EXO	60.8	77.4	1E-177	ref	ALK-EXO	*C. occidentalis* granulovirus
GVORF108	NP_148910.1	HELICASE-2	62.5	78.4	0	ref	ORF126 HELICASE-2	*C. pomonella* granulovirus
GVORF109	YP_654527.1	hypothetical protein COGV_gp106	38.2	59.2	5E-41	ref	hypothetical protein COGV_gp106	*C. occidentalis* granulovirus
GVORF110	NP_148915.1	lef-8	74.3	85.9	0	ref	ORF131 lef-8	*C. pomonella* granulovirus
GVORF111	AAT77801.1	unknown	42.5	64.2	2E-16	gb	unknown	*C. anachoreta* granulovirus
GVORF112	NP_148917.1	ORF112 similar to *Cydia pomonella* GV ORF133	52.4	76.2	4E-13	ref	ORF133 similar to XcGV ORF170	*C. pomonella* granulovirus
GVORF113	NP_148918.1	ORF113 similar to *Cydia pomonella* GV ORF134	71.4	86.5	4E-73	ref	ORF134 similar to AcMNPV ORF53	*C. pomonella* granulovirus
GVORF114	YP_654531.1	hypothetical protein COGV_gp110	37.1	58.5	8E-56	ref	hypothetical protein COGV_gp110	*C. occidentalis* granulovirus
GVORF115	YP_654532.1	hypothetical protein COGV_gp111	57.4	72.2	3E-14	ref	hypothetical protein COGV_gp111	*C. occidentalis* granulovirus
GVORF116	AAT67151.1	vp1054	68.9	83.7	1E-140	gb	unknown	*C. anachoreta* granulovirus
GVORF117		unknown					No hit	No hit
GVORF118	NP_891974.1	fgf-3	57.5	73.3	1E-103	ref	hypothetical protein ClgVgp127	*C. leucotreta* granulovirus
GVORF119	YP_654536.1	Ecdysteroid UDP-glucosyltransferase	59	74.9	0	ref	egt	*C. occidentalis* granulovirus
GVORF120	NP_148927.1	ME53-like protein	57.9	78.1	1E-136	ref	ORF143 ME53	*C. pomonella* granulovirus

PiraGV-K ORFs are represented by the Query ID and the source ORFs are represented by Subject ID. Proteins from viral complete genomes were clustered by sequence similarity based on BLASTP pairwise alignments using the viral clusters of orthologous groups (VOG) approach. Pid - percent identity; Psi - percent similarity; Eval - Best hit E-value; Db - Databases.

Genomic sequence identity of PiraGV-K was studied against other known betabaculoviruses genomes, with a maximum identity of 99% with PiraGV-C (Table 2). The 1% difference was thought to be related to the presence of extra nucleotides in the intronic sequences of the PiraGV-K genome and not corresponded to any known ORF. The identity with other genomes was in the order of 42–58%, with greater identity with ChocGV (58.5%), CrleGV (55.78%) and CypoGV (55.6%) genome sequences. Of a total of 120 ORFs, only ORFs 9, 32, and 117 were considered unique to the PiraGV genome sequences of the Korean and Chinese strains. This represents 1.7% of the whole genome sequence. Also, 78 ORFs found in all betabaculoviruses sequences studied, have been called “core GV genes”. Based on gene function, PiraGV-K ORFs have been grouped into four functional categories (Table-3): transcription (10 genes), replication (11 genes), structural (25 genes), and auxiliary (15 genes), with 59 unrepresented in the annotation. The most conserved among the core set of genes was *granulin,* with 100% identity with PiraGV-C. We compared the identified PiraGV-C ODV associated proteins [11], with the structural proteins found in PiraGV-K and found that the ORFs complemented and matched each other. PiraGV-K-ORF 1 (*granulin*), ORF-14 (*odv-e18*), ORF-15 (*p49*), ORF-16 (*odv-e56*), ORF-17 (*p10*), ORF-39 (*odv-e66*), ORF-44 (*odv-e66a*), ORF-45 (*ubiquitin*), ORF-51 (*p74*), ORF-61 (*pif-1*), ORF-71 (*p6.9*), ORF-75 (*helicase-1*), ORF-81 (*vp39*), ORF-82 (*odv-e27*), ORF-85 (*vp91 capsid*), ORF-88 (*gp-41*), ORF-90 (*vlf-1*), ORF-93 (*DNA pol*), ORF-95 (*lef-3*), ORF-118 (*fgf-3*) and ORF-120 (*ME-53*) were also among the reported proteins in PiraGV-C. Other proteins common to both the PiraGV genomes were found to be hypothetical or unknown proteins.

**Table 2:** The granuloviruses genome used for the characterization of PiraGV-K.

GV	Refseq	GenBank	Length (nt)	GC (%)	Genes	Identity (%)	Completed	Nation	Ref.
AdorGV	NC_005038	AF547984	99,657	34	119	49	7/15/2003	UK	27
AgseGV	NC_005839	AY522332	131,680	37	132	46	4/9/2004	China	NCBI
ChocGV	NC_008168	DQ333351	104,710	32	116	58	6/19/2006	Canada	28
CrleGV	NC_005068	AY229987	110,907	32	129	56	8/13/2003	Germany	13
CypoGV	NC_002816	U53466	123,500	45	143	56	4/2/2001	UK	29
HearGV	NC_010240	EU255577	169,794	40	179	43	1/9/2008	USA	47
PhopGV	NC_004062	AF499596	119,217	35	130	52	7/1/2002	France	NCBI
PlxyGV	NC_002593	AF270937	100,999	40	121	44	10/29/2000	Japan	10
PsunGV	NC_013772	EU678671	176,677	39	183	45	1/30/2010	China	NCBI
SpliGV	NC_009503	DQ288858	124,121	38	136	42	5/30/2010	Korea	31
XecnGV	NC_002331	AF162221	178,733	40	181	43	6/7/2000	USA	11
PiraGV-C	NC_013797	GQ884143	108,592	33	120	99	2/11/2010	China	20

The ClanGV (GenBank ID: HQ116624) and EpapGV (GenBank ID: JN408834) sequence information have not been taken for the genome characterization of PiraGV-K due to their publication after the present work was completed.

**Table 3:** PiraGV-K genes grouped according to function.

Functional category	PiraGV-K (ORF)
**Transcription**	*pe-38* (23), *39K* (48), *lef-11* (49), *p47* (56), *lef-6* (65), *lef-5* (72*), lef-4* (80), *vlf*-*1* (90), *lef-9* (99), *lef*-*8* (110)
**Replication**	*ie-1* (6), *lef-2* (33), *lef-1* (60), *dbp* (66), *38.7 k* (59), *helicase-1* (75), *DNA polymerase* (93), *lef-3* (95), *DNA ligase* (102) *helicase-2* (108), me53 (120)
**Structural**	*granulin* (1), *pk-1* (3), *odv-e18* (14), *odv-e56* (16), *p10* (17), *pep-1* (20), *calyx/pep* (21), *pep-2* (22), *p13* (38), *odv-e66* (39), *pif-2* (40), *odv-e66a* (44), *p74* (51), *vp24* (58), *pif-1* (61), *p6.9* (71), *odv-e25* (76), *vp39* (81), *odv-e27* (82), *vp91* (85), *tlp20* (86), *gp41* (88), *desmoplakin* (94), *fp* (100), *vp1054* (116)
**Auxilliary**	*chitinase* (10), *cathepsin* (11), *gp37* (13), *p49* (15), *phosphohydrolase* (32), *metalloproteinase* (37), *ubiquitin* (45), *superoxide dismutase* (50), *Nudix hydrolase* (57), *fgf-1* (62), *iap* (79), *fgf-2* (105), *alk-exo* (107), *fgf-3* (118), *ecdysteroid UDP-glucosyl transferase* (119)
**Unknown**	ORF2, ORF4, ORF5, ORF7, ORF8, ORF9, ORF12, ORF18, ORF19, ORF24, ORF25, ORF26, ORF27, ORF28, ORF29, ORF30, ORF31, ORF34, ORF35, ORF36, ORF41, ORF42, ORF43, ORF46, ORF47, ORF51, ORF52, ORF53, ORF54, ORF55, ORF63, ORF64, ORF67, ORF68, ORF69, ORF70, ORF73, ORF74, ORF77, ORF78, ORF83, ORF84, ORF87, ORF89, ORF91, ORF92, ORF96, ORF97, ORF101, ORF103, ORF104, ORF106, ORF109, ORF111, ORF112, ORF113, ORF114, ORF115, ORF117

PiraGV-K ORF 98 encoded an inhibitor of apoptosis (*iap-5*) that seems to be betabaculovirus specific [21]. Also, PiraGV-K ORF 37 (homologous to Cypo46, Xecn40, and Plxy35) is likely a member of the stromelysin family within the matrix metalloproteinase (MMP) superfamily. It has been observed that this peptide is retained within infected cells until death, and subsequently is released into the body of the insect, causing proteolysis of tissues [4], [50]. The most conserved baculovirus gene is *polyhedrin*/*granulin*, the major component of occlusion bodies. Another conserved PiraGV-K structural gene was *odv-e25* (PiraGV-K, ORF 76), showing 80% amino acid identity to betabaculovirus homologs. In contrast, *p24* capsid (PiraGV-K-58, ORF 58), which encodes a protein associated with both ODV and BV [51], was found to be poorly conserved (60% average amino acid identity to other betabaculoviruses). The *p80*/*p87-*capsid gene was absent from the PiraGV-K genome, as with other betabaculovirus genomes. The putative *p10* (PiraGV-K, ORF17) gene showed similarities to three XecnGV ORFs (Xecn ORF 5, Xecn ORF 19, and Xecn ORF 83). Homologs of these three ORFs were found in PlxyGV (Plxy ORF 2, Plxy ORF 21, and Plxy ORF 50) and they were thus suggested to be *p10* homologs [4]. *p10* is implicated in occlusion body morphogenesis and disintegration of the nuclear body matrix, resulting in dissemination of OBs [52]. In NPV-infected cells, *p10* forms fibrillar structures in the nucleus and cytoplasm. PiraGV-K ORF 17 showed a significantly low identity 14%, with AcMNPV *p10*, and was smaller than its counterpart (104 vs 336 amino acids). A high sequence identity of 48% was noted with ClanGV *p10*, having 101 amino acid residues in relation to other betabaculoviruses.

The PiraGV-K genome did not encode the glycoprotein *gp64* that constitutes a major envelope fusion protein in AcMNPV, BmNPV, OpMNPV, and EppoMNPV [53], [54]. This protein thus appears to be unique to group I NPVs [55], [56]. Also, 19 *lef* genes have been found in AcMNPV genomes, and have been implicated in DNA replication and transcription [57]. Early baculovirus genes are transcribed by the host cell RNA polymerase II, but these are often transactivated by genes such as *ie-0, ie-1, ie-2,* and *pe38* [58]. Of these early baculovirus genes, the PiraGV-K genome contained only *ie-1* and it was found to be poorly conserved in comparison with other betabaculovirus genomes, except PiraGV-C. These genes have previously been reported to be poorly conserved among baculoviruses. The CypoGV and PhopGV genomes have been reported to have a *pe38*, consistent with PiraGV-K genome [21].

Six genes have been described as essential for baculovirus DNA replication: *lef-1*, *lef-2*, *lef-3*, *dnapol*, *helicase* and *ie-1* [59]. Homologs for all these necessary genes were found in the whole-genome of PiraGV-K with moderately conserved sequences. A PiraGV-K genome-wide scan suggested the absence of a *lef-7* homolog. Earlier reports suggested that *lef-7* was a group I NPV-specific gene, and stimulated transient DNA replication in AcMNPV and BmNPV [60], [61]. The PiraGV-K ORFs also encode a *DNA ligase* (PiraGV-K ORF 102) and a *helicase-2* (PiraGV-K ORF 108), in common with LdMNPV and other betabaculovirus genomes. The LdMNPV *DNA ligase* displays catalytic properties of a type-III DNA ligase [62]. Because the homologs of *helicase-2* and *DNA ligase* are involved in DNA repair and recombination [63], the PiraGV-K genes likely have similar functions. The PiraGV-K genome lacks large (rr1) and small (rr2) subunits of ribonucleotide reductase and deoxyuridyltriphosphate (dUTPase) genes, that may account for the loss of enzymatic functions during facilitation of virus replication in non-dividing cells, where dNTP pathways are inactive. The lack of these genes has also been noted in alphabaculoviruses, such as AcMNPV, BmNPV, HaSNPV, HzSNPV, and EppoMNPV and other betabaculoviruses, such as PlxyGV and XecnGV [4], [5], [22], [51], [63], [64]. Late transcription genes, including *lef 4–6*, *8–11*, *39K*, *p47,* and *vlf-1* [65] have been found among the PiraGV-K ORFs, except a *lef-10* homolog. The most conserved PiraGV-K *lef* homolog was *lef-8*, while *lef-6* was the most poorly conserved. It has been understood that the GV *lef-6* genes are smaller than the NPV *lef-6* genes (86–102 amino acids *vs* 138–187 amino acids) and were reported in the XecnGV genome [5].

*Chitinase* [66] and *cathepsin* were present as auxiliary genes in the PiraGV-K genome. These genes have been identified in almost all the baculoviruses completely sequenced to date, except PlxyGV [4] and AdorGV [19]. The protein products encoded by these genes provide selective advantages in the breakdown of insect tissues at the end of infection and the release of OBs to the environment, which then spread horizontally [67]. The lack of the same in the cases of the PlxyGV and AdorGV genomes may account for the infected larvae not lysing at the end of infection; this may lead to the spread of viral infection by discharging large amounts of virus from their posterior ends. PiraGV-K ORF 50 corresponded to superoxide dismutase (*sod*), a well-conserved gene in baculoviruses. Among the betabaculoviruses, it was not reported in the SpliGV genome, although it is known in other betabaculoviruses. Although, SOD functions as an endogenous antioxidant, its proper function in baculoviruses remains unknown. Gene deletion studies conducted in AcMNPV did not show any deleterious effect [68], although it may be predicted that SOD may protect OBs from superoxide radicals generated by exposure to sunlight in the environment.

PiraGV-K ORF 45 corresponded to a ubiquitin protein, which have been identified in all baculoviruses sequenced to date, although it was found fused to *gp37* as a single ORF in SpltMNPV [48]. Apart from *polyhedrin* and *granulin* [69], it is also one of the most highly conserved genes in the baculovirus genome, with 73% average amino acid identity to betabaculovirus homologs. Interestingly, the homolog of viral ubiquitin has not been reported in AcMNPV-ODV or HearNPV-ODV, but is known in AcMNPV-BV [70]. *Per os* infectivity factors (*pif*), another highly conserved gene, involved in oral infectivity of baculovirus ODV, has been characterized from almost all baculovirus genomes sequenced so far. We identified ORF 61, corresponding to *pif-1,* and ORF 16, corresponding to *ODV-E56,* also known as *pif-5* [71] in the PiraGV-K genome. Although *pif*-1 and *p74* (ORF 51 in the PiraGV-K genome) have been proposed to form structural components of the ODV envelope and may regulate infectivity of OBs, *pif-5* is not an essential protein for binding and fusion of ODV or virus replication [72], [73]. Additionally, the PiraGV-K genome was found to contain three putative fibroblast growth factors (*fgf*), represented by ORFs 62, 105, and 118. These *fgfs* contained the *fgf* superfamily domains, as determined by a conserved family domain search with the BLAST program. No *enhancin* homolog was found in PiraGV-K genome and is consistent with the absence of the same in the AdorGV, CypoGV and PlxyGV genomes. In contrast to the above betabaculovirus genomes, four *enhancin* homologs were reported in XecnGV, two in LdMNPV, and one in MacoNPV. Enhancin functions in disrupting the insect peritrophic membrane, and facilitates the initiation of infection [74]. PiraGV-K ORF 13 corresponded to the *gp37* homolog (spindling acting as enhancing factor) that was shown to be absent from the AdorGV, AgseGV, ChocGV, CrleGV, PhopGV, PlxyGV, and SpliGV genomes, although the ORF was reported in the CypoGV, HearGV, PsunGV, XecnGV, and PiraGV-C genomes.

Furthermore, PiraGV-K was found to lack a *conotoxin-like (ctl)* homolog, as reported in the BmNPV, SeMNPV, HaSNPV, AdorGV, CypoGV, and PlxyGV genomes, although a *ctl* homolog has been identified in the genome of XecnGV. The ORF contains a six-cysteine motif similar to that in chitin-binding proteins [75]. A gene encoding protein kinase 1 (*pk-1*; PiraGV-K ORF 3) was also identified in the whole-genome sequence of PiraGV-K; this may be involved in the regulation of the phosphorylation status of viral and host proteins during infection. Two members of the *iap* genes, corresponding to *iap* (PiraGV-K ORF 79) and *iap5* (PiraGV-K ORF 98), were also identified in the PiraGV-K genome. Although the *p35* with antiapoptotic activity has been identified previously in the AcMNPV, BmNPV, and SpltMNPV genomes, it is absent from betabaculovirus genomes. The *iap* homologs generally contain two baculovirus IAP repeats (BIP) [76], that are associated with binding to apoptosis-inducing proteins [77], and a C-terminal zinc finger-like (RING) Cys/His motif [78]. The *iap-5* appears to be GV-specific, and all betabaculoviruses sequenced to date have *iap-5*. PiraGV-K ORF 94 is a homolog of Plxy ORF 94, named *desmoplakin* because it shows similarity to an internal region of a human *desmoplakin*, an essential constituent of intracellular junctions [4]. Baculovirus-repeated ORFs (*bro*) have not been seen in the PiraGV-K genome, although truncated versions have been observed in CpGV [21]. These repeats are more conspicuously present in many baculoviruses (1 and 16 copies), although their function is unclear, with the possibility of binding to DNA.

Two uncharacterized ORFs were also identified in the whole genome sequence of PiraGV-K and PiraGV-C, indicated as PiraGV-K ORF 9 and PiraGV-K ORF 117.

## Conclusions

There has been a significant increase in the number of whole-genome sequencing projects using the shotgun method, but traditional mapped clone methods using BAC, cosmid, and fosmid libraries remain an important intermediate layer for hybrid sequencing strategies. With a view towards advancing the whole-genome sequencing strategies of infectious viruses, we adopted a method for the construction of a fosmid library of virus mixed with the infected host and further screening only the viral genomic library. The method overcomes the often-difficult need to culture and purify viruses by traditional methods of genome analysis and reduces the difficulties in obtaining starting material than would be necessary if starting with the purification of virus particles from inclusion bodies. The viral DNA is recovered in amounts sufficient for classical genome sequencing, without recourse to the use of automated high-throughput NGS technology. Thus, the analysis of the genome of PiraGV-K by the novel method of electrophoretic separation provides significant advances towards analysis of other infectious viruses.

## Supporting Information

Table S1 **Analysis and homology search of PiraGV-K ORFs.** The PiraGV-K ORFs have been analyzed for homology using representative granulovirus genomes such as *Adoxophyes orana* granulovirus (AdorGV), *Agrotis segetum* granulovirus (AgseGV), *Choristoneura occidentalis* granulovirus (ChocGV), *Cryptophlebia leucotreta* granulovirus (CrleGV), *Cydia pomonella* granulovirus (CypoGV), *Helicoverpa armigera* granulovirus (HearGV), *Phthorimaea operculella* granulovirus (PhopGV), *Pieris rapae* granulovirus-Chinese isolate (PiraGV-C), *Plutella xylostella* granulovirus (PlxyGV), *Pseudaletia unipuncta* granulovirus (PsunGV), *Spodoptera litura* granulovirus (SpliGV) and *Xestia c-nigrum* granulovirus (XecnGV). Pid and Psi refers to percent identity and percent similarity.
(DOCX)
