# Supplementary material for: Analysis of the Genome of a Korean Isolate of the Pieris rapae Granulovirus Enabled by Its Separation from Total Host Genomic DNA by Pulse-Field Electrophoresis
Source: PLoS One. 2013 Dec 31;8(12):e84183. doi: 10.1371/journal.pone.0084183 (PMC3877225; doi:10.1371/journal.pone.0084183)
Supplement: Table S1 — Analysis and homology search of PiraGV-K ORFs. The PiraGV-K ORFs have been analyzed for homology using representative granulovirus genomes such as Adoxophyes orana granulovirus (AdorGV), Agrotis segetum granulovirus (AgseGV), Choristoneura occidentalis granulovirus (ChocGV), Cryptophlebia leucotreta granulovirus (CrleGV), Cydia pomonella granulovirus (CypoGV), Helicoverpa armigera granulovirus (HearGV), Phthorimaea operculella granulovirus (PhopGV), Pieris rapae granulovirus-Chinese isolate (PiraGV-C), Plutella xylostella granulovirus (PlxyGV), Pseudaletia unipuncta granulovirus (PsunGV), Spodoptera litura granulovirus (SpliGV) and Xestia c-nigrum granulovirus (XecnGV). Pid and Psi refers to percent identity and percent similarity. (DOCX) [file pone.0084183.s001.docx]

| **PiraGV-K ORF** | **Position** | **Length (AA)** | **AdorGV** | | | **AgseGV** | | | **ChocGV** | | | **CrleGV** | | | **CypoGV** | | | **HearGV** | | | **PhopGV** | | | **PiraGV-C** | | | **PlxyGV** | | | **PsunGV** | | | **SpliGV** | | | **XecnGV** | | | |
| --- | --- | --- | --- | --- | --- | --- | --- | --- | --- | --- | --- | --- | --- | --- | --- | --- | --- | --- | --- | --- | --- | --- | --- | --- | --- | --- | --- | --- | --- | --- | --- | --- | --- | --- | --- | --- | --- | --- | --- |
|  |  |  | **ORF** | **Pid** | **Psi** | **ORF** | **Pid** | **Psi** | **ORF** | **Pid** | **Psi** | **ORF** | **Pid** | **Psi** | **ORF** | **Pid** | **Psi** | **ORF** | **Pid** | **Psi** | **ORF** | **Pid** | **Psi** | **ORF** | **Pid** | **Psi** | **ORF** | **Pid** | **Psi** | **ORF** | **Pid** | **Psi** | **ORF** | **Pid** | **Psi** | | **ORF** | **Pid** | **Psi** |
| 1 | 1 → 744 | 247 | 1 | 84.3 | 89.9 | 1 | 77.4 | 87.9 | 1 | 82.3 | 91.5 | 1 | 82.7 | 89.9 | 1 | 82.7 | 90.3 | 1 | 75.8 | 88.7 | 1 | 81.5 | 89.5 | 1 | 96.8 | 96.8 | 5 | 77 | 89.9 | 1 | 75.8 | 88.7 | 1 | 76.3 | 87.1 | | 1 | 75.8 | 88.7 |
| 2 | 741 ← 1154 | 137 | 2 | 37.9 | 54.7 | 2 | 54.5 | 72.7 | 2 | 61.9 | 73 | 2 | 53.4 | 63.8 | 2 | 50.8 | 60 | 2 | 38.3 | 61.7 | 2 | 44.8 | 74.1 | 2 | 99.3 | 99.3 |  |  |  |  |  |  | 2 | 44.6 | 66.1 | | 2 | 38.3 | 61.7 |
| 3 | 1153 → 1989 | 278 | 3 | 58.1 | 79.1 | 3 | 52.7 | 69.6 | 3 | 67 | 83.3 | 3 | 63.4 | 80.8 | 3 | 58.1 | 75.8 | 3 | 45.5 | 64.3 | 3 | 63.4 | 82.2 | 3 | 99.6 | 100 | 7 | 50.7 | 70.9 | 3 | 46.5 | 63.9 | 3 | 49.5 | 65 | | 3 | 45 | 63.7 |
| 4 | 2031 ← 2597 | 188 | 4 | 39.8 | 57 | 6 | 47.3 | 66.1 | 5 | 56.7 | 77 | 4 | 60.1 | 77.1 | 4 | 65.4 | 81.4 | 6 | 39.6 | 59.3 | 4 | 61.8 | 77.4 | 4 | 99.5 | 99.5 | 9 | 23.8 | 49.7 | 6 | 37.4 | 59.9 | 5 | 46.2 | 63.4 | | 7 | 39.6 | 59.9 |
| 5 | 2587 → 2826 | 79 | 5 | 40 | 57.1 | 7 | 26.9 | 53.7 | 6 | 48.7 | 60.3 | 5 | 39.7 | 50.7 | 5 | 39.7 | 53.4 |  |  |  | 5 | 35.1 | 56.8 | 5 | 81 | 81 | 10 | 35.6 | 60 |  |  |  |  |  |  | |  |  |  |
| 6 | 2965 ← 4347 | 460 | 6 | 48 | 69.6 | 8 | 39.3 | 61 | 7 | 55.1 | 76.1 | 6 | 51.8 | 70.4 | 7 | 51.4 | 70.3 | 8 | 32 | 53.1 | 6 | 42.6 | 61.2 | 6 | 92.4 | 93 | 11 | 30.1 | 50.8 | 8 | 32.2 | 55.1 | 7 | 31.8 | 54.1 | | 9 | 32.2 | 54.3 |
| 7 | 4393 → 4953 | 186 | 7 | 49.2 | 68.1 | 9 | 35.9 | 54.5 | 8 | 58.1 | 75.3 | 7 | 53.5 | 72.2 | 8 | 52.1 | 71.6 | 9 | 32.8 | 57.9 | 7 | 46.8 | 65.5 | 7 | 99.5 | 99.5 | 12 | 33.5 | 57.2 | 9 | 33 | 59.8 | 8 | 32.4 | 51 | | 10 | 32.8 | 58.5 |
| 8 | 4990 ← 5295 | 101 | 8 | 46 | 69 | 10 | 48 | 67 | 9 | 63.4 | 69.3 | 8 | 59.4 | 75.2 | 9 | 61.4 | 76.2 | 10 | 48 | 65 | 8 | 59.2 | 78.9 | 8 | 86.1 | 87.1 | 13 | 50.5 | 63.4 | 10 | 48 | 66 | 9 | 51 | 67 | | 11 | 48 | 66 |
| 9 | 5369 → 5620 | 83 |  |  |  |  |  |  |  |  |  |  |  |  |  |  |  |  |  |  |  |  |  | 9 | 98.8 | 98.8 |  |  |  |  |  |  |  |  |  | |  |  |  |
| 10 | 5711 ← 7471 | 586 |  |  |  | 32 | 60.9 | 75.3 |  |  |  |  |  |  | 10 | 63.4 | 76.4 | 5 | 62.8 | 76.4 |  |  |  | 10 | 96.2 | 96.4 |  |  |  | 108 | 61.3 | 75.2 |  |  |  | | 103 | 64 | 77.6 |
| 11 | 7546 → 8565 | 339 |  |  |  | 31 | 62.1 | 80.7 |  |  |  | 11 | 70.1 | 86.5 | 11 | 69.2 | 82.8 |  |  |  |  |  |  | 11 | 100 | 100 |  |  |  | 65 | 52.5 | 71.8 |  |  |  | | 58 | 48.5 | 68.8 |
| 12 | 8570 → 8785 | 71 |  |  |  |  |  |  |  |  |  | 12 | 51.1 | 68.9 | 12 | 64.9 | 86.5 |  |  |  |  |  |  | 12 | 100 | 100 |  |  |  |  |  |  |  |  |  | |  |  |  |
| 13 | 8853 ← 9575 | 240 |  |  |  |  |  |  |  |  |  |  |  |  | 13 | 59.8 | 75.2 | 9 | 44.3 | 64.1 |  |  |  | 13 | 99.2 | 100 |  |  |  | 110 | 45.2 | 64.4 |  |  |  | | 107 | 45.1 | 64.6 |
| 14 | 9771 → 9878 | 92 | 10 | 46.5 | 50.7 |  |  |  | 12 | 40.8 | 45.1 | 13 | 42 | 47.8 | 14 | 42.3 | 50.7 |  |  |  | 12 | 40.3 | 48.6 | 14 | 69 | 69 |  |  |  |  |  |  |  |  |  | |  |  |  |
| 15 | 9875 ← 11257 | 460 | 11 | 49.7 | 70.3 | 12 | 50.9 | 69.8 | 13 | 63.8 | 80 | 14 | 62.1 | 79.3 | 15 | 61.8 | 79.4 | 12 | 41.7 | 62 | 13 | 38.1 | 56.2 | 15 | 100 | 100 | 15 | 41.7 | 61.5 | 12 | 40.4 | 62.2 | 11 | 43.5 | 63.5 | | 13 | 41.4 | 62.3 |
| 16 | 11527 ← 12591 | 354 | 12 | 65.5 | 80.3 | 15 | 57.8 | 72.3 | 14 | 72.8 | 85.5 | 17 | 67 | 81.2 | 18 | 69.8 | 81.2 | 14 | 55.5 | 71.9 | 16 | 69.4 | 80.9 | 16 | 86.4 | 86.4 | 17 | 65.3 | 77.1 | 14 | 55.1 | 71.9 | 13 | 53.1 | 70.5 | | 15 | 53.4 | 72.3 |
| 17 | 12628 ← 12942 | 104 |  |  |  |  |  |  |  |  |  |  |  |  |  |  |  |  |  |  |  |  |  | 17 | 75 | 75 | 3 | 34 | 49 |  |  |  |  |  |  | |  |  |  |
| 18 | 12958 ← 13359 | 133 | 14 | 29.5 | 46.2 |  |  |  |  |  |  |  |  |  |  |  |  |  |  |  |  |  |  | 18 | 88.7 | 88.7 | 4 | 30.5 | 44.5 |  |  |  |  |  |  | |  |  |  |
| 19 | 13543 → 13779 | 78 |  |  |  |  |  |  | 16 | 49 | 78.4 | 19 | 37.5 | 71.4 | 19 | 48.7 | 65.4 |  |  |  | 17 | 41.9 | 59.5 | 19 | 100 | 100 | 18 | 34.7 | 67.3 |  |  |  |  |  |  | |  |  |  |
| 20 | 13806 ← 14420 | 204 | 16 | 47.9 | 59.9 | 18 | 70.9 | 76.7 | 17 | 52.5 | 64 | 20 | 54.6 | 66.3 | 20 | 50.4 | 61 | 16 | 43.5 | 61 | 19 | 47.3 | 58.7 | 20 | 93.1 | 93.1 | 21 | 70.2 | 79.8 | 16 | 46.1 | 62.3 | 15 | 47.6 | 61.4 | | 17 | 44.5 | 61 |
| 21 | 14545 → 15567 | 340 | 17 | 59.4 | 70.1 | 19 | 54.3 | 67.1 | 18 | 65.5 | 73.6 | 23 | 65 | 73.6 | 22 | 67.1 | 77.2 | 18 | 46.5 | 58.5 | 20 | 54.1 | 64.2 | 21 | 95.6 | 95.6 | 22 | 52.8 | 66.6 | 18 | 47.9 | 58.9 | 19 | 44 | 58.5 | | 19 | 46.5 | 58.5 |
| 22 | 15584 → 16021 | 145 | 18 | 48.3 | 61.7 | 20 | 61.8 | 74.8 | 19 | 64.6 | 76.4 | 24 | 63.6 | 78 | 23 | 67.4 | 76.5 | 17 | 55.7 | 69.5 | 21 | 59.7 | 68.2 | 22 | 100 | 100 | 24 | 54.3 | 68.5 | 17 | 57.3 | 69.5 | 18 | 48.9 | 63.4 | | 18 | 56.5 | 70.2 |
| 23 | 16295 ← 17158 | 287 |  |  |  |  |  |  |  |  |  | 25 | 26.5 | 47.9 | 24 | 27.2 | 43.9 |  |  |  | 24 | 33.3 | 46.9 | 23 | 92.7 | 94.1 |  |  |  |  |  |  |  |  |  | |  |  |  |
| 24 | 17993 → 19282 | 429 | 21 | 24.7 | 41.7 | 23 | 33.3 | 50 | 22 | 38 | 60.3 | 28 | 33.9 | 57.6 | 29 | 35.7 | 55.1 |  |  |  |  |  |  | 24 | 95.6 | 96.3 |  |  |  |  |  |  |  |  |  | |  |  |  |
| 25 | 19668 → 20204 | 178 | 22 | 29.3 | 58 | 24 | 25.2 | 46.5 |  |  |  | 29 | 39.9 | 64.6 | 30 | 41.5 | 63.6 |  |  |  | 26 | 38.2 | 58.8 | 25 | 100 | 100 |  |  |  |  |  |  |  |  |  | |  |  |  |
| 26 | 20297 → 22066 | 589 | 23 | 44.4 | 65 | 25 | 53.9 | 75.1 | 23 | 67.7 | 82.4 | 30 | 62.6 | 80.2 | 31 | 63.6 | 79.4 | 26 | 34.5 | 56.7 | 27 | 61.9 | 78.8 | 26 | 97.8 | 98 | 27 | 42.8 | 63.4 | 26 | 34.3 | 57.6 | 24 | 42.2 | 62.4 | | 27 | 35 | 57.8 |
| 27 | 22240 → 23268 | 342 |  |  |  |  |  |  |  |  |  |  |  |  |  |  |  |  |  |  |  |  |  | 27 | 78.1 | 78.4 |  |  |  |  |  |  |  |  |  | |  |  |  |
| 28 | 23450 ← 24400 | 316 | 25 | 36.4 | 57 | 27 | 33.8 | 48.1 | 24 | 46.7 | 62.1 | 32 | 40.8 | 63.9 | 33 | 38.6 | 56.7 | 28 | 24.5 | 49.7 | 29 | 39.6 | 60.4 | 28 | 91.3 | 91.3 | 29 | 29.9 | 45.2 | 28 | 23.8 | 50 | 26 | 25.9 | 48.8 | | 29 | 25.8 | 49.1 |
| 29 | 24408 ← 24977 | 189 |  |  |  | 28 | 51.6 | 68.1 |  |  |  | 33 | 63 | 77.2 | 34 | 64.4 | 74.5 | 29 | 43.9 | 65.2 | 30 | 58.5 | 71.3 | 29 | 100 | 100 |  |  |  | 29 | 46 | 67.4 |  |  |  | | 30 | 38.1 | 65.3 |
| 30 | 24988 → 25560 | 190 | 26 | 49.1 | 69.8 | 29 | 47.7 | 63.8 | 26 | 56.2 | 76.3 | 34 | 54 | 70.6 | 35 | 56.9 | 70.7 | 30 | 47.2 | 71.7 | 31 | 52 | 68.7 | 30 | 92.1 | 92.1 | 30 | 49.4 | 66.9 | 30 | 46.1 | 68.3 | 27 | 50 | 66.7 | | 32 | 47.2 | 71.7 |
| 31 | 25593 → 25907 | 104 | 29 | 48.5 | 66 | 34 | 50 | 75 | 28 | 62 | 75 | 36 | 63.4 | 78.2 | 39 | 62.7 | 79.4 | 32 | 47.5 | 70 | 34 | 52.9 | 70.2 | 31 | 88.5 | 88.5 | 32 | 43 | 78.5 | 32 | 47.5 | 70 | 31 | 38 | 59.5 | | 34 | 47.5 | 70 |
| 32 | 25945 ← 26529 | 194 |  |  |  |  |  |  |  |  |  |  |  |  |  |  |  |  |  |  |  |  |  | 32 | 99 | 100 |  |  |  |  |  |  |  |  |  | |  |  |  |
| 33 | 26711 → 27229 | 172 | 32 | 40.5 | 59.5 | 35 | 36 | 56.6 | 29 | 59.5 | 75.6 | 38 | 52.7 | 74 | 41 | 54.1 | 70.6 | 33 | 37.6 | 52.4 | 37 | 46 | 64.9 | 33 | 94.8 | 95.3 | 33 | 39 | 56.1 | 33 | 37.1 | 51.2 | 32 | 37.1 | 54.5 | | 35 | 37.6 | 52.4 |
| 34 | 27231 → 27479 | 82 | 33 | 40.2 | 58.5 | 36 | 37.8 | 52.7 | 30 | 52.4 | 65.9 | 39 | 48.8 | 62.2 | 42 | 48.8 | 65.9 | 34 | 42.1 | 59.6 | 38 | 43.4 | 65.1 | 34 | 100 | 100 |  |  |  | 34 | 40.4 | 57.9 | 33 | 40.3 | 58.1 | | 36 | 42.1 | 59.6 |
| 35 | 27497 ← 27841 | 114 |  |  |  |  |  |  | 31 | 45.9 | 67.6 | 40 | 34.5 | 62.8 | 43 | 33 | 54.1 |  |  |  | 39 | 44.1 | 58.6 | 35 | 97.4 | 98.2 |  |  |  |  |  |  |  |  |  | |  |  |  |
| 36 | 27917 ← 28369 | 150 | 36 | 35.6 | 59.8 | 40 | 30.1 | 50 | 32 | 47.7 | 65.8 |  |  |  | 45 | 28.3 | 53.6 |  |  |  |  |  |  | 36 | 91.3 | 91.3 | 35 | 28.6 | 44.3 |  |  |  |  |  |  | |  |  |  |
| 37 | 28424 ← 29722 | 432 | 37 | 33.6 | 52.1 | 41 | 35.7 | 50.5 | 33 | 48.5 | 64.1 | 43 | 46.3 | 64.5 | 46 | 45.4 | 61.9 | 38 | 32.3 | 48.4 | 41 | 41.4 | 59.8 | 37 | 91.9 | 91.9 | 36 | 28.7 | 45.3 | 38 | 33.5 | 50.7 | 37 | 31.4 | 49.4 | | 40 | 31.6 | 49 |
| 38 | 29737 → 30537 | 266 | 38 | 54.3 | 75.7 | 42 | 56.4 | 72.3 | 34 | 58.3 | 75.6 | 44 | 56.3 | 74.3 | 47 | 59.7 | 75.4 | 40 | 50.6 | 71.3 | 42 | 55.3 | 72.2 | 38 | 100 | 100 | 37 | 49.8 | 66.4 | 41 | 50.2 | 69.7 | 38 | 48.5 | 64.7 | | 43 | 50.9 | 71.7 |
| 39 | 30503 → 32488 | 661 | 28 | 32.6 | 49 | 33 | 22.2 | 44.4 | 27 | 29.8 | 47.2 | 35 | 29.2 | 44.7 | 37 | 35.3 | 54.4 | 50 | 32.3 | 51 | 33 | 29.9 | 45.8 | 39 | 91.4 | 91.5 | 31 | 36.4 | 50.3 | 156 | 27.8 | 45 | 125 | 20.7 | 44.4 | | 149 | 32.3 | 51 |
| 40 | 32505 → 33632 | 375 | 39 | 60.3 | 77.1 | 43 | 52.4 | 68.8 | 35 | 68.8 | 80.4 | 45 | 69.3 | 79.6 | 48 | 69.8 | 80.7 | 42 | 52.8 | 65.5 | 44 | 61.9 | 78.2 | 40 | 93.6 | 93.8 | 38 | 54.8 | 69.5 | 43 | 52.5 | 65.8 | 39 | 55.4 | 68.6 | | 45 | 52.3 | 65 |

| **PiraGV-K ORF** | **Position** | **Length (AA)** | **AdorGV** | | | **AgseGV** | | | **ChocGV** | | | **CrleGV** | | | **CypoGV** | | | **HearGV** | | | **PhopGV** | | | **PiraGV-C** | | | **PlxyGV** | | | **PsunGV** | | | **SpliGV** | | | **XecnGV** | | |
| --- | --- | --- | --- | --- | --- | --- | --- | --- | --- | --- | --- | --- | --- | --- | --- | --- | --- | --- | --- | --- | --- | --- | --- | --- | --- | --- | --- | --- | --- | --- | --- | --- | --- | --- | --- | --- | --- | --- |
|  |  |  | **ORF** | **Pid** | **Psi** | **ORF** | **Pid** | **Psi** | **ORF** | **Pid** | **Psi** | **ORF** | **Pid** | **Psi** | **ORF** | **Pid** | **Psi** | **ORF** | **Pid** | **Psi** | **ORF** | **Pid** | **Psi** | **ORF** | **Pid** | **Psi** | **ORF** | **Pid** | **Psi** | **ORF** | **Pid** | **Psi** | **ORF** | **Pid** | **Psi** | **ORF** | **Pid** | **Psi** |
| 41 | 33624 ← 33833 | 69 |  |  |  |  |  |  |  |  |  | 46 | 30.4 | 67.4 |  |  |  |  |  |  | 45 | 37 | 67.4 | 41 | 78.3 | 78.3 |  |  |  |  |  |  |  |  |  |  |  |  |
| 42 | 33845 → 36256 | 803 | 42 | 29.2 | 47.9 | 45 | 29.8 | 46.5 |  |  |  | 47 | 39.1 | 55.4 | 50 | 39.2 | 56.1 | 44 | 28.4 | 47.3 | 46 | 45.3 | 61.4 | 42 | 93.6 | 94.3 | 39 | 30.6 | 61.1 | 45 | 28.7 | 48.8 | 40 | 27.4 | 46 | 47 | 26.5 | 46 |
| 43 | 36259 ← 36885 | 208 | 43 | 78 | 92.5 | 46 | 63 | 76.9 | 37 | 74.8 | 85 | 50 | 78 | 88 | 52 | 78.1 | 86.4 | 45 | 63.6 | 81 | 47 | 80.8 | 89.6 | 43 | 100 | 100 | 41 | 61 | 75.7 | 47 | 62 | 81 | 41 | 63.8 | 78.4 | 50 | 63.6 | 81.5 |
| 44 | 37056 ← 39380 | 774 | 28 | 49.9 | 67.7 | 33 | 36.7 | 57.1 | 27 | 56 | 70.9 | 35 | 55.9 | 72.3 | 37 | 58.9 | 73.8 | 50 | 42.3 | 59.4 | 33 | 57.6 | 73.7 | 44 | 98.7 | 99.2 | 31 | 51.8 | 68.4 | 156 | 41.2 | 58.1 | 125 | 22.7 | 42.5 | 149 | 42.8 | 59.6 |
| 45 | 39485 ← 39772 | 95 | 45 | 75.3 | 88.2 | 47 | 76.8 | 89.5 | 39 | 89.2 | 93.5 | 52 | 88.2 | 90.8 | 54 | 81.9 | 90.4 | 47 | 89.5 | 94.7 | 49 | 81.3 | 92.3 | 45 | 100 | 100 | 43 | 74.7 | 83.2 | 50 | 86.8 | 94.7 | 45 | 81.4 | 88.4 | 52 | 89.5 | 94.7 |
| 46 | 39857 → 40921 | 354 | 46 | 56.5 | 75.3 | 48 | 57.1 | 74.1 | 40 | 65.8 | 80.8 | 53 | 61.1 | 75.5 | 55 | 54.7 | 71.1 | 48 | 43.3 | 62.3 | 50 | 47 | 67.3 | 46 | 100 | 100 | 44 | 49.6 | 65.2 | 51 | 44.5 | 61 | 46 | 47.6 | 68.7 | 53 | 43.3 | 62.3 |
| 47 | 40927 → 41118 | 63 | 47 | 37.7 | 65.6 | 49 | 59 | 76.9 | 41 | 58.7 | 76.1 | 54 | 59.5 | 81.1 | 56 | 53.1 | 76.6 |  |  |  | 51 | 51.3 | 79.5 | 47 | 100 | 100 |  |  |  |  |  |  | 47 | 38.1 | 63.5 |  |  |  |
| 48 | 41167 ← 42030 | 287 | 49 | 36.1 | 54.6 | 51 | 43.7 | 60.5 | 42 | 44.2 | 61.4 | 55 | 61.8 | 79.4 | 57 | 39.3 | 54.1 | 50 | 25.3 | 50 | 52 | 38.3 | 53.5 | 48 | 93 | 93.4 | 46 | 29.9 | 52.9 | 53 | 26.3 | 50.3 | 48 | 27.8 | 46.8 | 55 | 25.1 | 49.7 |
| 49 | 42008 ← 42304 | 98 | 50 | 47.8 | 68.5 | 52 | 51.6 | 69.9 | 43 | 65.2 | 79.3 | 56 | 58.2 | 76.9 | 58 | 64.1 | 79.3 | 51 | 51.1 | 67 | 53 | 57.8 | 75.9 | 49 | 100 | 100 | 47 | 48.9 | 66.7 | 54 | 53.3 | 70.7 | 49 | 46 | 64.4 | 56 | 52.1 | 69.1 |
| 50 | 42345 ← 42815 | 156 | 51 | 56.7 | 69.3 | 54 | 62.6 | 78.1 | 44 | 67.5 | 79 | 57 | 68.9 | 79.5 | 59 | 71.2 | 82.6 | 63 | 62.3 | 72.6 | 54 | 67.3 | 82.7 | 50 | 99.4 | 100 | 48 | 58.3 | 70.2 | 64 | 62.3 | 74.7 |  |  |  | 68 | 62.3 | 73.3 |
| 51 | 43098 ← 45131 | 677 | 53 | 50.5 | 65.9 | 56 | 51.8 | 66.3 | 46 | 60.6 | 73.3 | 58 | 58.2 | 71.9 | 60 | 58.7 | 71.7 | 72 | 42.7 | 59.7 | 55 | 56.3 | 70.4 | 51 | 93.5 | 93.5 | 50 | 52.1 | 68.4 | 77 | 43.5 | 59.9 | 56 | 27.6 | 44.1 | 77 | 43 | 60.5 |
| 52 | 45424 → 46011 | 195 |  |  |  |  |  |  |  |  |  |  |  |  |  |  |  |  |  |  | 64 | 32.2 | 48.9 | 52 | 99 | 99.5 |  |  |  |  |  |  |  |  |  |  |  |  |
| 53 | 46348 ← 46830 | 160 |  |  |  |  |  |  |  |  |  |  |  |  |  |  |  |  |  |  |  |  |  | 53 | 76.5 | 77.8 |  |  |  |  |  |  |  |  |  |  |  |  |
| 54 | 46838 ← 47437 | 199 | 56 | 54.6 | 72.4 | 57 | 57.4 | 73.6 | 48 | 61.6 | 77.8 |  |  |  |  |  |  |  |  |  | 59 | 59.3 | 74.9 | 54 | 96 | 98.5 |  |  |  |  |  |  |  |  |  |  |  |  |
| 55 | 47662 ← 48105 | 147 |  |  |  | 59 | 63 | 87 | 49 | 78 | 90 | 60 | 71.7 | 88.3 | 62 | 70 | 90 | 73 | 58.2 | 72.7 | 60 | 68.9 | 86.9 | 55 | 91.2 | 91.2 | 51 | 58.9 | 78.6 | 78 | 60.4 | 73.6 | 57 | 54.8 | 72.6 |  |  |  |
| 56 | 48147 → 49346 | 399 | 58 | 58.5 | 74.4 | 60 | 57 | 71.6 | 50 | 69.8 | 80.9 | 61 | 69.2 | 81.9 | 68 | 65.1 | 81.7 | 74 | 54.9 | 71.4 | 61 | 62.3 | 76.3 | 56 | 97.2 | 97.5 | 52 | 54.5 | 72.2 | 79 | 55 | 70.3 | 58 | 54.9 | 71.6 | 78 | 54.9 | 71.4 |
| 57 | 49389 → 50054 | 221 | 59 | 75.3 | 87.4 | 61 | 68.3 | 84.4 | 51 | 76.7 | 90 | 62 | 77.8 | 90.5 | 69 | 76.9 | 88.7 | 77 | 65.6 | 82.4 | 62 | 73.8 | 86.7 | 57 | 99.5 | 99.5 | 53 | 63.6 | 81.3 | 82 | 66.1 | 82.8 | 59 | 62.1 | 80.8 | 79 | 65.6 | 82.8 |
| 58 | 50246 → 50899 | 217 | 60 | 53 | 71.1 | 62 | 52.5 | 73.1 | 52 | 57.1 | 75 | 63 | 60.8 | 78.9 | 71 | 59.3 | 75.4 | 78 | 41.1 | 59.5 | 63 | 47.5 | 65.4 | 58 | 92.2 | 92.2 | 54 | 55.2 | 69.9 | 83 | 41.9 | 63.8 | 60 | 43.5 | 65.8 | 80 | 42.8 | 62.7 |
| 59 | 50930 ← 51454 | 174 |  |  |  | 63 | 43.8 | 61.8 | 54 | 39.9 | 56.6 | 65 | 39.8 | 61.4 | 73 | 47.1 | 62.4 | 79 | 29 | 52.7 | 65 | 42.5 | 67.8 | 59 | 85.1 | 85.6 | 55 | 51.7 | 64.4 | 84 | 30.7 | 52.3 | 61 | 33.7 | 61.8 | 81 | 32.3 | 53.8 |
| 60 | 51435 ← 52136 | 233 | 62 | 56.6 | 74.5 | 64 | 57.4 | 73.5 | 55 | 68.7 | 82.4 | 66 | 65 | 82.9 | 74 | 65.7 | 81.1 | 80 | 49.4 | 66.7 | 66 | 53.3 | 72.1 | 60 | 100 | 100 | 56 | 50 | 69.1 | 85 | 51.3 | 68.5 | 62 | 51.1 | 68.9 | 82 | 49.8 | 67.1 |
| 61 | 52179 → 53804 | 541 | 63 | 54.2 | 71.1 | 65 | 37.2 | 54.4 | 56 | 64.2 | 78.9 | 67 | 61.9 | 77.4 | 75 | 61.7 | 79.3 | 82 | 46.7 | 66.9 | 67 | 52.3 | 67.7 | 61 | 96.7 | 96.9 | 8 | 51.5 | 69 | 87 | 45.9 | 65.5 | 64 | 44.3 | 63.9 | 84 | 45.8 | 66.3 |
| 62 | 53920 ← 54600 | 226 | 64 | 39.8 | 60.2 | 66 | 32 | 50.2 | 57 | 58.9 | 77.2 | 68 | 50 | 68.4 | 76 | 48 | 66.7 | 83 | 26.8 | 47.4 | 69 | 46.5 | 64.6 | 62 | 100 | 100 | 57 | 36.2 | 55.6 | 88 | 30 | 47.6 | 66 | 37.1 | 54.6 | 85 | 25.8 | 44 |
| 63 | 54656 ← 54955 | 99 |  |  |  |  |  |  | 58 | 37 | 54 | 69 | 34 | 46 | 77 | 32.7 | 51 |  |  |  | 70 | 32.9 | 53.7 | 63 | 86.9 | 87.9 |  |  |  |  |  |  |  |  |  |  |  |  |
| 64 | 55073 → 55555 | 160 | 66 | 38.2 | 52.8 | 67 | 38.6 | 56.8 | 59 | 43.9 | 56.1 | 70 | 39.5 | 52.9 | 79 | 43.1 | 57.8 | 85 | 32.5 | 50.4 | 71 | 40.3 | 54.8 | 64 | 100 | 100 | 60 | 48.9 | 57.4 | 90 | 34.5 | 48.8 | 68 | 43.5 | 58.7 | 87 | 31.9 | 51.3 |
| 65 | 55615 ← 55929 | 104 | 67 | 41.3 | 62.7 | 68 | 45.5 | 61.8 | 60 | 50.7 | 72.6 | 71 | 47.4 | 65.8 | 80 | 50 | 68.2 | 86 | 45.2 | 61.3 | 72 | 40.3 | 68.1 | 65 | 86.5 | 86.5 | 61 | 42.9 | 66.1 | 91 | 50 | 60.3 | 69 | 39.7 | 67.6 | 88 | 43.5 | 61.3 |
| 66 | 55982 ← 56827 | 281 | 68 | 55 | 78.7 | 69 | 28.8 | 47.6 | 61 | 66.8 | 82.8 | 72 | 54.9 | 74 | 81 | 54 | 73.2 | 87 | 27.7 | 50.8 | 73 | 36.3 | 54.3 | 66 | 100 | 100 | 62 | 27.2 | 51.2 | 92 | 27.4 | 51.4 | 70 | 24.7 | 47.8 | 89 | 27.7 | 51.3 |
| 67 | 57026 ← 57607 | 193 |  |  |  | 71 | 22.5 | 41.5 | 63 | 32.5 | 50 | 73 | 30.8 | 47.4 | 82 | 29.3 | 41.4 |  |  |  | 74 | 24 | 40.8 | 67 | 92.2 | 92.7 |  |  |  |  |  |  |  |  |  |  |  |  |
| 68 | 57638 → 58807 | 389 | 69 | 65.7 | 80.9 | 72 | 60.6 | 76.1 | 64 | 75.8 | 88.7 | 74 | 72.9 | 85.6 | 83 | 78 | 87.3 | 90 | 52.8 | 70.5 | 75 | 67 | 79.9 | 68 | 96.7 | 96.7 | 64 | 48.7 | 71.4 | 95 | 53.4 | 71 | 73 | 50.5 | 71.1 | 91 | 53.1 | 71.2 |
| 69 | 58862 → 59191 | 109 | 70 | 48.6 | 63.6 | 73 | 36.3 | 50 | 65 | 41.5 | 65.1 | 75 | 49.5 | 58.7 | 84 | 55.9 | 63.7 | 91 | 33.3 | 49.4 |  |  |  | 69 | 82.6 | 82.6 |  |  |  | 96 | 32.9 | 49.4 | 74 | 34.7 | 50.7 | 92 | 33.3 | 49.4 |
| 70 | 59254 → 60378 | 374 | 71 | 60.9 | 79.6 | 74 | 58.7 | 77.7 | 66 | 70.6 | 85.6 | 76 | 66.9 | 81.3 | 85 | 69 | 83.3 | 92 | 49.4 | 69.2 | 77 | 52.9 | 69.3 | 70 | 100 | 100 | 67 | 46 | 69.8 | 97 | 49.3 | 68.5 | 75 | 51 | 70.4 | 93 | 48.8 | 69 |
| 71 | 60402 → 60578 | 58 |  |  |  |  |  |  |  |  |  |  |  |  |  |  |  |  |  |  |  |  |  |  |  |  |  |  |  |  |  |  |  |  |  |  |  |  |
| 72 | 60638 ← 61375 | 245 | 73 | 62.6 | 76.8 | 76 | 54.6 | 69.9 | 68 | 74.8 | 83.5 | 78 | 72.7 | 83.1 | 87 | 71.8 | 83.7 | 94 | 57 | 71.9 | 79 | 69.4 | 80.2 | 72 | 100 | 100 | 70 | 56.4 | 73.7 | 99 | 54.9 | 70.4 | 76 | 61.9 | 74.6 | 95 | 58.8 | 73.5 |
| 73 | 61322 → 62227 | 301 | 74 | 65.6 | 77.9 | 77 | 52.1 | 67.3 | 69 | 70.8 | 84.2 | 79 | 62.1 | 77.1 | 88 | 62.5 | 74.2 | 95 | 49.5 | 65.3 | 80 | 63.9 | 77.9 | 73 | 100 | 100 | 71 | 46.4 | 66 | 100 | 49.2 | 66.7 | 77 | 50.8 | 68.4 | 96 | 48.8 | 65 |
| 74 | 62255 ← 62740 | 161 | 75 | 55.6 | 69.9 | 78 | 51.1 | 71.9 | 70 | 69.6 | 83.7 | 80 | 61.9 | 79.9 | 89 | 68.3 | 80.6 | 96 | 52.9 | 74.3 | 81 | 50.9 | 68.3 | 74 | 90.1 | 90.1 | 72 | 41.7 | 70.9 | 101 | 53.7 | 75 | 79 | 48.5 | 65.4 | 97 | 52.2 | 74.3 |
| 75 | 62724 → 66125 | 1133 | 76 | 53 | 69.6 | 80 | 41.4 | 58.5 | 71 | 63.8 | 78.6 | 81 | 56.3 | 74.1 | 90 | 55.7 | 74.2 | 97 | 40.1 | 58.4 | 82 | 44.7 | 64 | 75 | 98.2 | 98.2 | 73 | 38.8 | 57.7 | 102 | 40.5 | 59.1 | 80 | 40.7 | 58.2 | 98 | 40.3 | 59 |
| 76 | 66215 ← 66859 | 214 | 77 | 59 | 72.8 | 81 | 68.4 | 82.5 | 72 | 65.7 | 80.3 | 82 | 73.4 | 81.8 | 91 | 71.5 | 82.7 | 98 | 52.7 | 68.6 | 83 | 64.5 | 77.6 | 76 | 87.4 | 87.9 | 75 | 55.9 | 71.8 | 103 | 51.6 | 69.9 | 81 | 57.1 | 73.7 | 99 | 52.3 | 69.1 |
| 77 | 66937 ← 67419 | 160 | 78 | 40.5 | 65.2 | 82 | 51 | 69 | 73 | 54.4 | 79.7 | 83 | 56 | 76.1 | 92 | 60.4 | 79.9 | 99 | 43.3 | 61.8 | 84 | 55.1 | 73.4 | 77 | 98.8 | 100 | 76 | 44.4 | 65 | 104 | 41.1 | 62 | 82 | 42.2 | 62.3 | 100 | 47.1 | 66.9 |
| 78 | 67479 → 68234 | 251 | 79 | 62.4 | 78.4 | 83 | 60.2 | 77.3 | 74 | 68 | 85.6 | 84 | 70.4 | 85.6 | 93 | 68.5 | 88 | 100 | 55.8 | 73.3 | 85 | 64.1 | 83.3 | 78 | 99.6 | 100 | 77 | 61.4 | 77.1 | 105 | 55 | 73.7 | 83 | 54.4 | 68.8 | 101 | 53.8 | 73.3 |
| 79 | 68247 ← 68900 | 217 | 89 | 33.3 | 58.2 | 53 | 39.5 | 54.1 | 84 | 38.2 | 57.2 | 85 | 37 | 60.2 | 94 | 38.3 | 59.6 | 139 | 27.3 | 45.3 | 86 | 39.1 | 56.4 | 79 | 99.5 | 100 | 98 | 26.4 | 40.5 | 143 | 25.7 | 42.7 | 16 | 38.4 | 58.1 | 137 | 27.3 | 45.9 |
| 80 | 68905 ← 70341 | 478 | 80 | 49.6 | 68.4 | 85 | 47.5 | 63.7 | 75 | 58.9 | 72.2 | 86 | 55.9 | 71.5 | 95 | 55.2 | 70.4 | 112 | 41.7 | 59.3 | 87 | 52.3 | 68.9 | 80 | 94.1 | 94.1 | 79 | 40.3 | 58.9 | 114 | 42.1 | 59.4 | 86 | 44.1 | 60.1 | 110 | 41.6 | 58.8 |

| **PiraGV-K ORF** | **Position** | **Length (AA)** | **AdorGV** | | | **AgseGV** | | | **ChocGV** | | | **CrleGV** | | | **CypoGV** | | | **HearGV** | | | **PhopGV** | | | **PiraGV-C** | | | **PlxyGV** | | | **PsunGV** | | | **SpliGV** | | | **XecnGV** | | |
| --- | --- | --- | --- | --- | --- | --- | --- | --- | --- | --- | --- | --- | --- | --- | --- | --- | --- | --- | --- | --- | --- | --- | --- | --- | --- | --- | --- | --- | --- | --- | --- | --- | --- | --- | --- | --- | --- | --- |
|  |  |  | **ORF** | **Pid** | **Psi** | **ORF** | **Pid** | **Psi** | **ORF** | **Pid** | **Psi** | **ORF** | **Pid** | **Psi** | **ORF** | **Pid** | **Psi** | **ORF** | **Pid** | **Psi** | **ORF** | **Pid** | **Psi** | **ORF** | **Pid** | **Psi** | **ORF** | **Pid** | **Psi** | **ORF** | **Pid** | **Psi** | **ORF** | **Pid** | **Psi** | **ORF** | **Pid** | **Psi** |
| 81 | 70409 → 71269 | 286 | 81 | 66.6 | 80.3 | 86 | 41.2 | 55.7 | 76 | 73.4 | 84.3 | 87 | 66.3 | 81.6 | 96 | 65.4 | 81.3 | 113 | 39.4 | 54.6 | 88 | 53 | 66.3 | 81 | 100 | 100 | 80 | 32.6 | 48.4 | 115 | 39.7 | 55.4 | 87 | 38.9 | 52.9 | 111 | 39.4 | 55.6 |
| 82 | 71329 → 72189 | 286 | 82 | 64.9 | 83.5 | 87 | 49.7 | 70.3 | 77 | 71.4 | 86.4 | 88 | 66.4 | 84.6 | 97 | 68.2 | 86.1 | 114 | 46.4 | 64.7 | 89 | 57.3 | 78.7 | 82 | 100 | 100 | 81 | 42.4 | 68.4 | 116 | 47.8 | 63.7 | 88 | 49.5 | 69.3 | 112 | 46.7 | 64.7 |
| 83 | 72358 ← 73509 | 383 | 83 | 41.2 | 65 | 88 | 31.1 | 49.2 | 78 | 49.5 | 67.7 | 90 | 45.9 | 63.4 | 99 | 44.5 | 65.2 | 116 | 27.8 | 47.4 | 92 | 29.6 | 50.9 | 83 | 91.9 | 91.9 | 83 | 34.1 | 51.6 | 117 | 27.1 | 46 | 89 | 27.9 | 46.3 | 113 | 28.5 | 47.3 |
| 84 | 73580 → 73873 | 97 |  |  |  | 90 | 65.4 | 84.6 |  |  |  | 91 | 49.2 | 59 | 100 | 42.6 | 54.4 | 119 | 32.5 | 42.9 | 93 | 46.2 | 56.4 | 84 | 86.6 | 86.6 | 84 | 58.3 | 83.3 | 120 | 33.7 | 43.5 | 90 | 73.1 | 92.3 | 116 | 32.5 | 42.9 |
| 85 | 73857 ← 75803 | 648 | 85 | 37.2 | 60.7 | 92 | 33.9 | 53.9 | 80 | 45.4 | 61.9 | 92 | 45.1 | 64.2 | 101 | 43.5 | 60.2 | 121 | 33.6 | 56.2 | 94 | 45.8 | 64.1 | 85 | 95.3 | 95.5 | 85 | 43.4 | 64.2 | 122 | 30.7 | 52.7 | 92 | 33.8 | 58.3 | 118 | 33.2 | 55.8 |
| 86 | 75772 → 76320 | 182 | 86 | 37.3 | 57.3 | 93 | 26.7 | 49.2 | 81 | 42.6 | 67.6 | 93 | 39 | 54.2 | 102 | 34.4 | 43.9 |  |  |  | 95 | 36.3 | 54.9 | 86 | 100 | 100 | 86 | 33 | 46.4 |  |  |  | 93 | 22.6 | 44.7 |  |  |  |
| 87 | 76295 → 76885 | 196 | 87 | 75.6 | 90.6 | 94 | 47.1 | 63.1 | 82 | 66.7 | 75.9 | 94 | 64.5 | 74.9 | 103 | 60.6 | 73.4 | 123 | 48.3 | 65.2 | 96 | 58.4 | 69 | 87 | 81.1 | 81.1 | 87 | 55.2 | 66.9 | 124 | 48.9 | 66.3 | 94 | 48.1 | 64.1 | 120 | 48.3 | 65.2 |
| 88 | 76854 → 77714 | 286 | 88 | 48.3 | 67.6 | 95 | 60.5 | 78 | 83 | 67.7 | 83.9 | 95 | 71.3 | 85.7 | 104 | 73.8 | 88.1 | 124 | 49.5 | 68.9 | 97 | 64.6 | 81.1 | 88 | 100 | 100 | 88 | 47.3 | 68.9 | 125 | 51.2 | 69.8 | 95 | 54 | 75.1 | 121 | 49.5 | 68.9 |
| 89 | 77774 → 78067 | 97 | 90 | 30.1 | 49.5 |  |  |  | 85 | 46.3 | 58.9 | 96 | 40 | 59 | 105 | 41.2 | 53.6 | 125 | 28.6 | 48 | 98 | 42.2 | 53.9 | 89 | 83.5 | 83.5 |  |  |  |  |  |  |  |  |  |  |  |  |
| 90 | 78018 → 79145 | 375 | 91 | 70.1 | 81.5 | 97 | 64 | 81.7 | 86 | 69.6 | 84.1 | 97 | 74.9 | 88.3 | 106 | 75.5 | 86.6 | 126 | 54.3 | 71.4 | 99 | 71 | 85.9 | 90 | 97.6 | 97.6 | 90 | 47.4 | 68.6 | 127 | 53.8 | 72.1 | 97 | 57.5 | 75.7 | 123 | 54.3 | 71.4 |
| 91 | 79170 → 79424 | 84 | 92 | 50 | 59.5 | 99 | 34.2 | 48.1 | 88 | 53.6 | 60.7 | 98 | 45.1 | 53.7 | 107 | 51.2 | 57.1 | 128 | 38 | 46.8 | 100 | 45.9 | 54.1 | 91 | 66.7 | 66.7 | 92 | 32.9 | 45.6 | 129 | 36.7 | 46.8 | 99 | 38.8 | 52.9 | 125 | 38 | 46.8 |
| 92 | 79436 → 79888 | 150 | 93 | 58.5 | 78.9 | 100 | 49.3 | 71.6 | 89 | 65.3 | 88 | 99 | 64.7 | 83.3 | 108 | 60.7 | 80.7 | 129 | 30.3 | 59.2 | 101 | 62.3 | 82.8 | 92 | 99.3 | 100 | 93 | 37.6 | 59.1 | 130 | 29.6 | 60.5 | 100 | 36.9 | 66.4 | 126 | 30.3 | 59.2 |
| 93 | 79967 ← 83107 | 1046 | 94 | 58.1 | 73.4 | 101 | 57.2 | 73.5 | 90 | 69.1 | 79.8 | 101 | 61.3 | 76.5 | 111 | 64.8 | 76.4 | 134 | 50.7 | 68 | 103 | 63.7 | 75.6 | 93 | 96.8 | 97 | 94 | 49.3 | 66.4 | 138 | 50 | 67.5 | 101 | 50.8 | 67.3 | 132 | 50.7 | 68 |
| 94 | 83106 → 85331 | 741 | 95 | 26.2 | 42.9 | 102 | 44.2 | 64 | 91 | 31.6 | 49.6 | 102 | 33.1 | 49.6 | 112 | 31 | 48.4 | 135 | 47.7 | 67.4 | 104 | 30.4 | 49.6 | 94 | 87.3 | 87.3 | 95 | 47.1 | 65.9 | 139 | 46.5 | 65.1 | 102 | 43.8 | 66.2 | 133 | 47.7 | 66.3 |
| 95 | 85507 ← 86526 | 339 | 96 | 47.1 | 66.8 | 103 | 25.8 | 47.5 | 92 | 54.9 | 76.1 | 103 | 45.9 | 65.2 | 113 | 44.3 | 65.5 | 136 | 30.4 | 52.1 | 105 | 28.5 | 50.8 | 95 | 100 | 100 | 96 | 25.4 | 48.3 | 140 | 26.9 | 50.2 | 103 | 21.2 | 43.6 | 134 | 29.3 | 51.2 |
| 96 | 86498 → 86875 | 125 | 97 | 59.5 | 73.6 | 104 | 52.5 | 77 | 93 | 70.4 | 89.6 | 104 | 65.8 | 86.7 | 114 | 68.1 | 86.6 | 137 | 40 | 68.8 | 106 | 53.8 | 81.5 | 96 | 100 | 100 | 97 | 45.8 | 71 | 141 | 41.6 | 70.4 | 104 | 47.9 | 71.9 | 135 | 40.9 | 69.1 |
| 97 | 86913 → 87431 | 172 | 98 | 32.9 | 57.1 | 105 | 34.3 | 52.7 | 94 | 43.1 | 62.8 | 105 | 34.3 | 55.4 | 115 | 33.3 | 54.9 |  |  |  | 107 | 44 | 61.1 | 97 | 91.3 | 91.9 |  |  |  | 142 | 25.3 | 43.5 | 105 | 26.9 | 44.4 |  |  |  |
| 98 | 87460 → 88278 | 272 | 99 | 50.4 | 66.3 | 106 | 49.3 | 60.2 | 95 | 63.9 | 78.4 | 106 | 61.6 | 75.3 | 116 | 60.9 | 73.4 | 139 | 31.8 | 50.2 | 108 | 55.9 | 73.3 | 98 | 97.1 | 97.4 | 99 | 40.2 | 56.5 | 143 | 31 | 48 | 106 | 36.9 | 52.7 | 137 | 31.1 | 50.5 |
| 99 | 88259 → 89740 | 493 | 100 | 65.9 | 81.7 | 107 | 67.9 | 82 | 96 | 74.8 | 86.6 | 107 | 73.3 | 84.8 | 117 | 74.9 | 85.9 | 140 | 64.8 | 79.8 | 109 | 71.7 | 85.7 | 99 | 97.6 | 97.6 | 100 | 65.1 | 80 | 145 | 64.8 | 79.7 | 107 | 65.4 | 78.3 | 139 | 64.8 | 79.8 |
| 100 | 89769 → 90221 | 150 | 101 | 60.3 | 77.3 | 108 | 63.3 | 79.6 | 97 | 69.5 | 83.4 | 108 | 67.3 | 87.6 | 118 | 62.7 | 81.4 | 141 | 47.5 | 70.9 | 110 | 62.2 | 83.9 | 100 | 100 | 100 | 101 | 55 | 75 | 146 | 44.8 | 65.7 | 109 | 54.4 | 70.1 | 140 | 48.2 | 70.9 |
| 101 | 90309 → 90830 | 173 |  |  |  |  |  |  | 98 | 42.3 | 59 | 109 | 40.8 | 63.4 | 119 | 43.1 | 60.6 | 167 | 41.5 | 64.8 | 111 | 38.9 | 54.1 | 101 | 100 | 100 | 66 | 28.2 | 51.8 |  |  |  |  |  |  | 169 | 40.9 | 65.4 |
| 102 | 90856 ← 92562 | 568 | 103 | 53.9 | 69.9 | 110 | 56.4 | 72.8 | 99 | 65.5 | 79.8 | 110 | 62.3 | 77.6 | 120 | 63.5 | 77.9 | 142 | 43.1 | 60.7 | 112 | 54.7 | 71.8 | 102 | 97.2 | 97.2 | 102 | 45.2 | 62.6 | 148 | 43.6 | 60.6 | 110 | 46.3 | 65 | 141 | 43.3 | 60.7 |
| 103 | 93005 → 93157 | 50 |  |  |  |  |  |  | 100 | 55.3 | 68.4 |  |  |  |  |  |  |  |  |  |  |  |  | 103 | 100 | 100 |  |  |  |  |  |  |  |  |  |  |  |  |
| 104 | 93210 → 93413 | 67 | 104 | 37.7 | 54.1 |  |  |  | 101 | 53.6 | 72.5 | 112 | 42.6 | 64.7 | 122 | 61.5 | 88.5 |  |  |  | 117 | 35.3 | 57.4 | 104 | 80.9 | 80.9 |  |  |  |  |  |  |  |  |  |  |  |  |
| 105 | 93436 ← 94737 | 433 | 105 | 24.6 | 46.3 | 113 | 33.4 | 54.8 | 102 | 43.1 | 62.1 | 113 | 41.5 | 61.4 | 123 | 42.1 | 61 | 145 | 26.6 | 43.1 | 116 | 39.5 | 57.6 | 105 | 92.4 | 92.6 | 105 | 24.7 | 44.2 | 151 | 26.6 | 41 | 116 | 30.5 | 48.2 | 144 | 27.2 | 43.8 |
| 106 | 94748 → 95062 | 104 | 106 | 35.8 | 57.9 |  |  |  | 103 | 54.8 | 76.3 | 114 | 50 | 75.6 | 124 | 57.6 | 80.4 |  |  |  | 115 | 63 | 85.9 | 106 | 100 | 100 |  |  |  |  |  |  |  |  |  |  |  |  |
| 107 | 95118 → 96317 | 399 | 107 | 43.6 | 65.1 | 115 | 49 | 68.7 | 104 | 58.8 | 74.4 | 115 | 55.4 | 73 | 125 | 59.1 | 73.6 | 146 | 41.8 | 60.8 | 114 | 55.9 | 75.6 | 107 | 96.2 | 96.2 | 107 | 45.9 | 65.2 | 152 | 43.7 | 61.8 | 117 | 42.5 | 61.8 | 145 | 41.3 | 60.8 |
| 108 | 96241 → 97581 | 446 | 108 | 44.9 | 65.5 | 116 | 49.8 | 68.3 | 105 | 56.1 | 71.5 | 116 | 58.1 | 76.9 | 126 | 62.5 | 78.4 | 147 | 48.1 | 68 | 113 | 61.4 | 76.9 | 108 | 99.8 | 100 | 108 | 49.5 | 66.3 | 153 | 48.5 | 67.3 | 118 | 50.5 | 68.2 | 146 | 49.3 | 68.2 |
| 109 | 97794 ← 98801 | 335 | 109 | 24 | 51.6 | 117 | 27.8 | 46.5 | 106 | 36.8 | 57.5 | 118 | 43 | 64.5 | 130 | 46.5 | 69.7 |  |  |  |  |  |  | 109 | 83.6 | 83.9 | 109 | 37.9 | 61.1 |  |  |  | 119 | 26.5 | 47.9 |  |  |  |
| 110 | 98855 ← 101497 | 880 | 110 | 66 | 77.6 | 118 | 66.6 | 78.1 | 107 | 72.9 | 83.7 | 119 | 72.3 | 82.6 | 131 | 73.2 | 84.1 | 149 | 59.9 | 74.6 | 121 | 69.5 | 80.6 | 110 | 97.8 | 97.8 | 110 | 62.8 | 75.5 | 155 | 60.7 | 74.7 | 121 | 62.3 | 74.2 | 148 | 60.1 | 74.3 |
| 111 | 101708 → 102043 | 111 |  |  |  | 120 | 35.5 | 64.5 |  |  |  | 120 | 32.4 | 51.4 | 132 | 41.9 | 62.9 | 163 | 34.9 | 54.2 |  |  |  | 111 | 99.1 | 100 |  |  |  | 169 | 32.5 | 50.6 | 122 | 34.9 | 48.2 | 165 | 39.6 | 62.3 |
| 112 | 102160 ← 102336 | 58 |  |  |  | 121 | 36.6 | 65.9 | 108 | 48.8 | 76.7 |  |  |  | 133 | 52.4 | 76.2 |  |  |  |  |  |  | 112 | 98.1 | 100 |  |  |  |  |  |  |  |  |  |  |  |  |
| 113 | 102335 → 102736 | 133 | 111 | 54.9 | 69.9 | 122 | 48.5 | 66.2 | 109 | 66.9 | 83.5 | 121 | 69.9 | 86.5 | 134 | 71.4 | 86.5 | 169 | 39.5 | 62 | 122 | 69.2 | 81.2 | 113 | 100 | 100 | 113 | 43.2 | 64 | 173 | 40.1 | 62 | 127 | 43.3 | 61.4 | 171 | 38 | 59.1 |
| 114 | 102729 ← 103700 | 323 | 113 | 35.9 | 52.5 | 124 | 33 | 48.6 | 110 | 37.2 | 59.4 | 122 | 29.5 | 48.5 | 135 | 41.4 | 55.7 | 170 | 34.1 | 45 | 123 | 35.4 | 52.2 | 114 | 96 | 96.3 |  |  |  | 174 | 31.8 | 45.7 | 128 | 34.1 | 48.8 | 172 | 34.1 | 45 |
| 115 | 103703 ← 103915 | 70 | 114 | 42.9 | 71.4 |  |  |  | 111 | 57.4 | 72.2 |  |  |  | 136 | 30.4 | 55.1 |  |  |  |  |  |  | 115 | 100 | 100 |  |  |  |  |  |  |  |  |  |  |  |  |
| 116 | 103996 → 104988 | 330 | 115 | 54.9 | 70.9 | 127 | 50.6 | 68.7 | 113 | 63 | 79.4 | 125 | 61.3 | 75.8 | 138 | 63.7 | 79 | 173 | 45.2 | 61.5 | 126 | 55 | 71.2 | 116 | 99.7 | 99.7 | 116 | 40.4 | 56.5 | 177 | 46.8 | 62.2 | 130 | 43.7 | 60.6 | 175 | 45.5 | 61.5 |
| 117 | 105003 → 105221 | 72 |  |  |  |  |  |  |  |  |  |  |  |  |  |  |  |  |  |  |  |  |  | 117 | 100 | 100 |  |  |  |  |  |  |  |  |  |  |  |  |
| 118 | 105297 → 106127 | 276 | 117 | 43.5 | 60.2 | 128 | 40.9 | 58.6 | 114 | 51.6 | 68.8 | 127 | 53 | 65.8 | 140 | 48.2 | 65.2 | 176 | 30.8 | 50.8 | 128 | 53.2 | 69.9 | 118 | 90.5 | 90.5 | 105 | 23.8 | 43.4 | 180 | 30 | 51.5 | 133 | 32.3 | 54.8 | 178 | 30.8 | 50.8 |
| 119 | 106140 ← 107528 | 462 | 118 | 39.8 | 61.1 | 129 | 53.7 | 71.6 | 115 | 59 | 74.9 | 128 | 56.4 | 74.6 | 141 | 56.6 | 73.8 |  |  |  | 129 | 51.3 | 69.1 | 119 | 99.6 | 99.8 | 119 | 44.1 | 60.6 |  |  |  |  |  |  |  |  |  |
| 120 | 107707 → 108618 | 303 | 119 | 43 | 62.6 | 131 | 36.4 | 55.8 | 116 | 54.3 | 68.1 | 129 | 48.7 | 67.2 | 143 | 52 | 70.9 | 178 | 31 | 50 | 130 | 45.2 | 64.5 | 120 | 92.1 | 92.4 | 1 | 37 | 54.5 | 182 | 28.1 | 49.4 | 134 | 32 | 50.7 | 180 | 30.3 | 50.3 |
